# *Hypericum perforatum* L. extract alleviates metabolic-associated fatty liver disease through inflammation, lipid metabolism and ferroptosis modulation: a multi-omics perspective

**DOI:** 10.1186/s13020-025-01248-1

**Published:** 2025-12-03

**Authors:** Peng Huang, Yunling Zhu, Yidan Qin, Jianguo Hu, Jiancheng Wang, Jian Qin

**Affiliations:** 1 Department of Traditional Chinese Medicine, The Seventh Affiliated Hospital, Sun Yat-Sen University, Shenzhen, 518107 China; 2 Scientific Research Center, The Seventh Affiliated Hospital, Sun Yat-Sen University, Shenzhen, 518107 China

**Keywords:** *Hypericum perforatum* L., Metabolic-associated fatty liver disease (MAFLD), Multi-omics, Inflammation oxidative stress-Lipid metabolism axis, Ferroptosis

## Abstract

**Background:**

*Hypericum perforatum* L. (Guan Ye Jin Si Tao, GYJST), commonly known as St. John’s wort, is a widely distributed medicinal plant across Europe and Asia. Preclinical studies have identified its therapeutic potential in both neurological and metabolic disorders. However, its impact on metabolic-associated fatty liver disease (MAFLD) remains unclear. This study comprehensively investigated the therapeutic effects of GYJST on MAFLD through both in vivo and in vitro experiments. Utilizing a multi-omics approach, the research elucidated the regulatory mechanisms of GYJST on ferroptosis, focusing on oxidative stress, lipid metabolism, and inflammatory response modulation. The findings provide valuable insights into the potential therapeutic applications of GYJST in managing MAFLD.

**Materials and methods:**

Liver assessments were systematically conducted to evaluate the therapeutic effects of GYJST on HFD-induced mice and PA-induced AML12 cells. Comprehensive histological analyses, including H&E, Masson, Sirius Red, Oil Red O, and F4/80 staining, were performed to assess the impact of GYJST on liver pathology. To elucidate the underlying mechanisms of GYJST, a multi-omics approach was employed, integrating network pharmacology, transcriptomics, proteomics, and metabolomics. Additionally, RT-qPCR, western blotting, and immunofluorescence techniques were utilized to validate the effects of GYJST.

**Results:**

GYJST effectively protects against liver injury by mitigating inflammation, oxidative stress, and lipid metabolism dysregulation. A total of 90 major compounds in GYJST were tentatively identified. Network pharmacology analysis revealed its multi-target, multi-pathway mechanisms of action. Integrative transcriptomic, metabolomic, and proteomic analyses consistently highlighted pathways associated with inflammatory responses, oxidative stress, and lipid metabolism. Mechanistically, GYJST suppresses systemic inflammation via the NF-κB/COX-2 signaling axis and alleviates oxidative stress and lipid accumulation through the Nrf2/PPARα/g pathway. Additionally, GYJST plays a crucial role in inhibiting ferroptosis, partly through Nrf2-mediated mechanisms.

**Conclusion:**

GYJST exerts multi-target therapeutic effects against MAFLD by concurrently regulating ferroptosis, oxidative stress, lipid metabolism, and inflammation through interconnected mechanisms. These findings establish GYJST as a promising multi-target therapeutic candidate for MAFLD treatment.

**Graphical Abstract:**

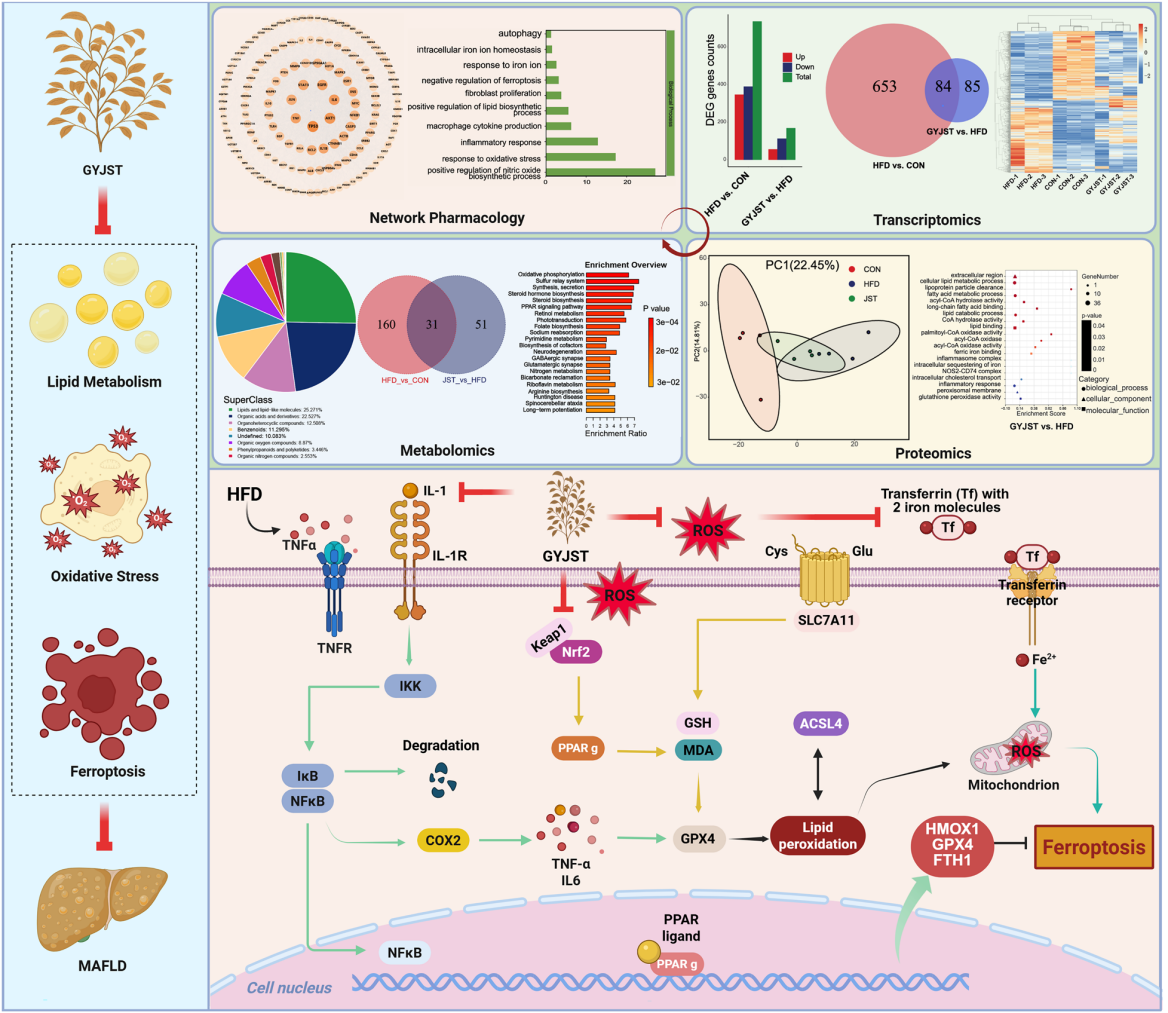

**Supplementary Information:**

The online version contains supplementary material available at 10.1186/s13020-025-01248-1.

## Introduction

Metabolic-associated fatty liver disease (MAFLD), introduced in 2020, represents a paradigm shift in understanding the relationship between non-alcoholic fatty liver disease (NAFLD) and systemic metabolic dysfunction [1]. With a global prevalence of 32.4%, MAFLD exhibits significant gender disparities, affecting 39.7% of males compared to 25.6% of females [2]. Geographically, Latin America bears the highest disease burden, followed by the Middle East, North Africa, Asia, and North America, while Western Europe maintains the lowest prevalence rates [3]. The athophysiology of MAFLD is characterized by complex hepatic lipid dysregulation, marked by enhanced de novo lipogenesis and triglyceride synthesis, coupled with impaired fatty acid oxidation and export mechanisms [4]. This imbalance leads to pathological lipid accumulation in hepatocytes. Disease progression is mediated through oxidative stress-induced lipid peroxidation, mitochondrial dysfunction, and activation of inflammatory cascades [5], driving the transition from simple steatosis to metabolic-associated steatohepatitis (MASH), hepatic fibrosis, and potentially hepatocellular carcinoma [6].

Current therapeutic strategies emphasize a multifaceted approach centered on lifestyle modifications and metabolic optimization. Key interventions include sustainable weight reduction, management of abdominal adiposity, improvement of insulin sensitivity, and prevention of metabolic syndrome and diabetes mellitus [7]. These measures aim to attenuate MASH progression and potentially induce fibrotic regression. Despite advancements in understanding MAFLD pathophysiology, significant challenges persist in establishing evidence-based therapeutic protocols and developing targeted pharmacological interventions tailored specifically for MAFLD management.

In the progression of MAFLD, ferroptosis is intricately linked with inflammation, lipid metabolism, and oxidative stress [8]. Ferroptosis, an iron-dependent form of cell death, is predominantly driven by lipid peroxidation [9]. In patients with MAFLD, hepatic lipid accumulation and metabolic abnormalities create a conducive environment for the onset of ferroptosis. Disrupted iron metabolism results in elevated levels of intracellular free iron, which generates reactive oxygen species (ROS) through the Fenton reaction, thereby exacerbating oxidative stress and lipid peroxidation, and ultimately triggering ferroptosis. Simultaneously, damage-associated molecular patterns (DAMPs) released during ferroptosis activate inflammatory pathways, further promoting hepatic inflammation and fibrosis. Additionally, dysfunction of the antioxidant system, such as glutathione peroxidase 4 (GPX4), is frequently observed in MAFLD, aggravating the vicious cycle of ferroptosis and oxidative stress. Consequently, ferroptosis plays a pivotal role in the pathological progression of MAFLD, interacting with inflammation, dysregulated lipid metabolism, and oxidative stress to collectively drive disease progression.

Multi-omics technologies have become indispensable in life sciences, enabling target identification, mechanistic exploration, and drug development for various diseases. Recent years have witnessed their exponential growth [10]. Transcriptomics and metabolomics are widely used for disease mechanism investigation, diagnosis, and progression monitoring, while proteomics primarily identifies differential protein expression across biological systems. The integration of these complementary approaches has gained momentum, as combined provides more comprehensive insights than single-omics studies [11, 12]. This study employed an integrated transcriptomics-proteomics-metabolomics approach to achieve a systematic understanding.

*Hypericum perforatum* L. (Guan Ye Jin Si Tao, GYJST), commonly known as St. John’s wort, is a widely distributed medicinal plant across Europe and Asia. Accumulating evidence has revealed its multifaceted pharmacological properties, demonstrating significant efficacy in both neurological and metabolic disorders. Beyond its well-established antidepressant effects [13], preclinical studies have identified its therapeutic potential in metabolic regulation, particularly through enhancing insulin sensitivity [14], improving glucose homeostasis, and promoting adipose thermogenesis in rodent models [15]. Additionally, emerging evidence suggests its hepatoprotective properties against cellular damage [16]. Nevertheless, the therapeutic potential and underlying mechanisms of GYJST in MAFLD remain poorly understood. Further investigation is warranted to elucidate its clinical efficacy and expand its therapeutic applications. Therefore, in this study, a mouse model of MAFLD was established through the administration of a high-fat diet (HFD). The protective effects and underlying mechanisms of GYJST were comprehensively investigated using an integrative approach that combined network pharmacology, transcriptomics, metabolomics, and proteomics analyses.

## Materials and methods

### Chemicals and reagents

Enzyme-linked immunosorbent assay (ELISA) kits for interleukin-1β (IL-1β), interleukin-6 (IL-6) and Tumor Necrosis Factor alpha (TNF-α) were purchased from Elabscience Industrial Co., Ltd (Wuhan, China). Palmitelaidic Acid (P799157) was purchased from Macklin. Primary antibodies of peroxisome proliferator-activated receptor g (PPARg) (Cat. sc-7273, 1:200), peroxisome proliferator-activated receptor alpha (PPARα) (Cat. sc-398394, 1:200), GPX4 (Cat.sc-166570, 1:200), transferrin receptor (TFRC)/CD71 (CD71, Cat. sc-65882, 1:200), anti-ACSL4 (Cat. sc-32282, 1:200) were from Santa Cruz Biotechnology, Inc. (Dallas, Texas, United States). The primary antibody of 4 hydroxynonenal (4-HNE, Cat. Ab46545, 1: 1000) was from Abcam Plc. (Cambridge, UK) and the anti-SLC7A11 (Cat. MA5-50633, 1: 500) was from Invitrogen (California, United States), the anti-COX2 (Cat:12282S, 1:1000), anti-NFkB p65 (Cat:4764, 1:1000), and anti-Nrf2 (Cat:12721S, 1:100) were from Cell Signaling Technology, Inc. These antibodies above were used for western blotting (WB). For immunofluorescence staining, goat anti-mouse IgG Fluor555-conjugated secondary (Cat. A-21422), and goat anti-mouse IgG Fluor488-conjugated secondary antibodies, (Cat.A11001), were obtained from Invitrogen (California, United States).

### Preparation of GYJST

GYJST (Lot: HX24D01) were purchased from the seventh affiliated hospital of Sun Yat-sen University (Shenzhen, Guangdong Province, China). Voucher specimens were deposited at the authors’ laboratory. The water extract of *Hypericum perforatum* L. was prepared according to a previous publication. Briefly, 1500 g of dried GYJST. Was soaked in 15 times its weight of distilled water for 1 h at ambient temperature, followed by 0.5 h reflux extractions. The resulting extraction solutions were pooled, concentrated to the desired volume, and then freeze-dried for 48 h to obtain the lyophilized powder of GYJST. The lyophilized powder yield was approximately 5.07%. The lyophilized powder was stored in different airtight packages before the studies.

### Chemical analysis of GYJST

The ultrahigh-performance liquid chromatography-high resolution mass spectrometry (UHPLC-HRMS) method was utilized to identify the chemical components in GYJST lyophilized powder. Briefly, the lyophilized GYJST powder was diluted proportionally with a 40% methanol solution and centrifuged at 16,000 rpm for 15 min. Subsequently, the supernatant was collected and stored at 4 °C.

GYJST were analyzed using a Vanquish UHPLC system (Thermo Scientific, Waltham, MA) equipped with HSS-T3 column (100 × 2.1 mm, 1.8 μm particles size; Waters) at a column compartment termperature of 35 °C. Mobile phase A is H_2_O + 0.1%formic acid and mobile phase B is acetonitrile + 0.1%formic acid (LC–MS grade solvents, Fisher chemical). Samples were separated with a flow rate of 0.3 mL/min using the following gradient: 1 min isocratic at 5% B, up to 98 B in 16 min, back to 5% B in 0.5 min and then 2.5 min isocratic at 5% B. Q-Exactive HFX mass spectrometer (Thermo Fisher Scientific, Bremen, Germany) was coupled to the UHPLC system. Mass spectra were acquired in both electrospray ionization (ESI) positive and negative modes using data-dependent acquisition (DDA) mode with a mass range of m/z 90–1300. The MS/MS spectra were obtained from the top10 most intense MS1 ions. The stepped normalized high-energy dissociation (HCD) collision energies (CEs) of 20, 40, and 60 units were used. Capillary temperature is 320 °C and probe heater temperature is 350 °C.

### Animal experiment

The animal studies were conducted in strict accordance with the ARRIVE guidelines and were approved by the Laboratory Animal Management and Use Committee of Shenzhen Top Biotechnology Co., LTD (Approval Number: TOP-LACUC-2021-0116). Adult male C57BL/6 J mice (8 weeks old) weighing between 18 and 21 g, were purchased from Zhuhai BesTest Bio-Tech Co. Ltd (Zhuhai, China). All mice were housed under controlled environmental conditions, including a temperature of 23–25 °C, a 12-h light/dark cycle, and humidity maintained at 55% ± 5%. The mice were acclimatized for at least 7 days with ad libitum access to standard laboratory chow and water.

**Fig. 1:**
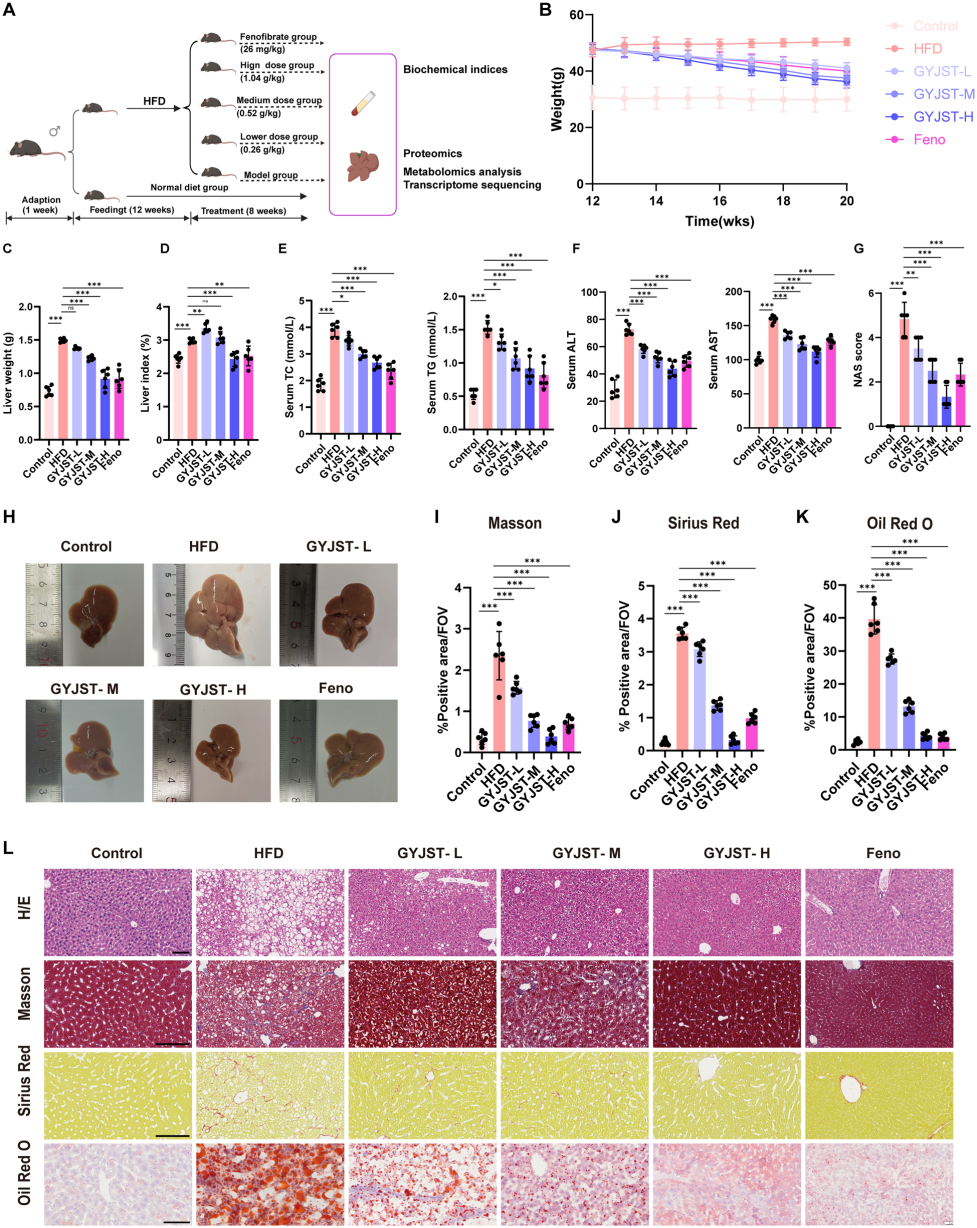
Effects of GYJST administration on phenotypes of MAFLD mice. **A** Animal experimental design. **B**–**D** Effects of GYJST and Feno on body weight, liver weight, and liver index in MAFLD mice. **E**, **F** Effects of GYJST and Feno on serum biochemical parameters. **G** Effects of GYJST and Feno on NAS score. **H** Morphological map of liver. **I**–**K** Quantification of the indicated stainings. **L** Representative immunohistochemistry images for H/E, Masson staining, Sirius red and Oil red O staining. Data are expressed as mean ± SEM. Statistical significance was calculated using one-way analysis of variance with Tukey’s multiple comparison test. n = 6, ^∗^p < 0.05, ^∗∗^p < 0.01 and ^∗∗∗^p < 0.001. ns, non-significant. Scale bars, 100 μm

The animal experimental design is shown in Fig. 1A. After a 1-week acclimatization period, six mice were randomly selected as controls, while the remaining animals were fed a HFD (60% kcal fat, Research Diets D12492) for 12 weeks to establish a mouse model of MAFLD [17, 18]. After successful modeling, the MAFLD mice were randomly assigned to five groups (n = 6 per group): the model groups (HFD), low-dose GYJST (GYJST-L), middle-dose GYJST (GYJST-M), high-dose GYJST (GYJST-H) groups and the positive control group.

The clinical dosage of GYJST (2 g) for a 70 kg adult is 0.26 g of crude drug per day, determined based on the body surface area index and the lyophilized powder yield of 5.07%. The equivalent low dosage for animal studies was calculated as 0.013 g/kg/day, which corresponds to the clinical dosage. To investigate the effects of GYJST on HFD-induced mice, varying doses of GYJST lyophilized powder (0.013, 0.026, and 0.052 g/kg/day) were administered orally to the mice. During the treatment period, control mice were fed a normal diet, while the other four groups were maintained on an HFD for 8 weeks. The positive control group received 26 mg/kg fenofibrate (Feno) [19, 20], and the control and model groups received saline. All treatments were delivered via gavage for a duration of 8 weeks.

Mice were weighed weekly, at the end of the treatment, the mice were anesthetized, and blood samples were collected for serum extraction through centrifugation (3500 rpm, 10 min, 4 °C), then stored at − 80 °C for biochemical analysis. The livers were carefully excised and weighed, and the hepatic index was calculated based on body weight. A portion of the liver tissue was collected for histopathological and omics analysis. The liver index was calculated as follows: Liver index = liver weight/body weight) × 100%.

### HE and oil red staining

Portions of the left lobes of the liver was immersed in 4% paraformaldehyde for 72 h, followed by paraffin embedding and sectioning into 3 μm slices. After rehydration and washing, liver tissue sections were stained with hematoxylin–eosin (H&E) and Masson’s trichrome stain. The stained tissue sections were photoimaged and observed under a light microscope (Digitalpathology section scanner, KFBIO, China, KF-PRO-020-HI).

### Biochemical assay

Serum total cholesterol (TC), triglyceride (TG), alanine transaminase (ALT) and aspartate aminotransferase (AST) were measured using automated biochemical instruments (Mindray CL-2000i). Lipid peroxidation malondialdehyde (MDA) assay kit ((Beyotime, S0131S) was applied to measure MDA of liver tissue and AML12 cells by thiobarbituric acid (TBA) method. A commercial glutathione (GSH) and oxidized glutathione disulfide (GSSG) assay (Beyotime, S0053) was used to evaluate the GSH/GSSH ratios of liver tissue. A nicotinamide adenine dinucleotide phosphate (NADPH) assay kit with WST-8 method (Beyotime, S0179) was employed to detect the NADPH in liver tissue. All of these experiments were performed according to the manuscript instructions. The serum levels of TNF-α, IL-1β and IL-6 were detected with ELISA kits from Elabscience Biotechnology Co., Ltd. (Wuhan, China) with specific protocols.

### NAFLD activity score (NAS)

Histological sections were quantitatively analyzed using the NAFLD Activity Score (NAS) system under blinded conditions. The NAS comprises three components: steatosis (scored 0–3), lobular inflammation (scored 0–3), and hepatocyte ballooning (scored 0–2), with a total possible score ranging from 0 to 8. According to standard diagnostic criteria, samples were categorized as: non-NASH (0–2 points), borderline NASH (3–4 points), or definitive NASH (5–8 points).

### Immunofluorescence (IF) staining

Tissue specimens were fixed in 4% paraformaldehyde (PFA) and embedded in optimal cutting temperature (OCT) compound for cryosectioning. Sections were incubated with primary antibodies at 4 °C overnight, followed by light-protected incubation with corresponding secondary antibodies for 1 h at room temperature. Antibody details are provided in chemicals and reagents. Fluorescence images were captured using either an LSM800 confocal microscope (Zeiss) or a Dragonfly CR-DFLY-202 2540 imaging system (Leica). Primary and secondary antibody information is shown in the Table S1.

### Network pharmacological analysis

#### Prediction of putative GYJST targets

The chemical constituents were identified via a combination of fragmentation pathway analysis, references comparison, and literature searches. The targets of screened compounds above were predicted and screened via applying the Integrative Pharmacology-based Research Platform of Traditional Chinese Medicine (TCMIP, Version 2.0, http://www.tcmip.cn/), SwissTargetPrediction tool (http://swisstargetprediction.ch/), CTD database (https://ctdbase.org/), and PharmMapper Server (https://www.lilab-ecust.cn/pharmmapper/).

#### MAFLD-associated therapeutic targets

Therapeutic targets for MAFLD treatment were identified from four databases. Genes with a gene-disease association evidence index of one in DisGeNET were selected. From GeneCards, genes with relevance scores above the median were chosen. Additionally, MAFLD-associated genes from the TTD, CTD and OMM databases were included.

#### Network analysis

To investigate the potential mechanism of GYJST on MAFLD, we first identified common targets by intersecting the candidate targets of GYJST with the therapeutic targets of MAFLD using a Venn diagram. These common targets were then imported into the STRING11.0 database (https://string-db.org/) for network topological analysis, with a combined score threshold of > 0.7. Key targets were subsequently selected based on topological parameters exceed twice the median value. Finally, the interaction network was visualized using Cytoscape software (Version 3.6.0).

#### Functional enrichment analysis

The results of the enrichment analyses for Gene Ontology (GO) biological processes, cellular components, molecular functions, and KEGG signaling pathways were generated by uploading the key targets to the DAVID platform (https://davidbioinformatics.nih.gov/home.jsp).

#### Liver transcriptome sequencing

Total RNA was extracted from liver tissue using TRIzol^®^ Reagent (Magen) following the manufacturer’s protocol. RNA quality was assessed by measuring the A260/A280 ratio using a Nanodrop ND-2000 system (Thermo Scientific, USA) and determining the RNA Integrity Number (RIN) with an Agilent Bioanalyzer 4150 system (Agilent Technologies, CA, USA). Only high-quality RNA samples (RIN > 8) were used for library preparation. Paired-end libraries were constructed using the ABclonal mRNA-seq Lib Prep Kit (ABclonal, China). Briefly, mRNA was isolated from 1 μg total RNA using oligo (dT) magnetic beads, fragmented, and reverse-transcribed into cDNA. Double-stranded cDNA was synthesized, adapter-ligated, and PCR-amplified. Libraries were purified (AMPure XP system) and quality-checked on the Agilent Bioanalyzer 4150 system. Sequencing was performed on an Illumina Novaseq 6000/MGISEQ-T7 platform. Raw data were processed and analyzed using an in-house bioinformatics pipeline at Shanghai Applied Protein Technology (Shanghai, China).

#### Liver proteomic analysis

Protein samples were prepared through a series of steps including denaturation, reduction, alkylation, tryptic digestion, and peptide purification. Briefly, tissues were homogenized, and 50 µL of lysis buffer was added, followed by mixing at 1000 rpm for 10 min. After cooling to room temperature, trypsin digestion buffer was introduced, and the samples were incubated at 37 °C with continuous shaking at 500 rpm for 2 h. Peptide purification was carried out using IST cartridges with the manufacturer-recommended wash buffer. Elution was performed twice with 100 µL of elution buffer, and the collected peptides were lyophilized using a SpeedVac concentrator. Subsequently, high-pH reversed-phase fractionation was conducted, followed by database searches. DDA raw data were processed using Spectronaut X (Biognosys AG, Switzerland) under default settings to generate a preliminary spectral library. For data-independent acquisition (DIA), raw data were analyzed using Spectronaut X with dynamic iRT-based retention time prediction and default parameters. Proteomic profiling services were provided by Shanghai Applied Protein Technology (Shanghai, China).

#### Liver metabolomics analysis

Following thawing at 4 °C, liver tissue was homogenized in a pre-cooled methanol/acetonitrile/water solution (2:2:1, v/v), ultrasonicated at low temperature for 30 min, and incubated at − 20 °C for 10 min. After centrifugation at 14,000 g (4 °C) for 20 min, the supernatant was lyophilized and reconstituted in acetonitrile/water (1:1, v/v) for analysis. Chromatographic separation was performed on a Vanquish UHPLC system equipped with a HILIC column (25 °C) using a gradient of (A) water containing 25 mM ammonium acetate and 25 mM ammonia and (B) acetonitrile at a flow rate of 0.5 mL/min. The gradient program was as follows: 0–0.5 min, 95% B; 0.5–7 min, 95–65% B; 7–8 min, 65–40% B; 8–9 min, 40% B; 9–9.1 min, 40–95% B; 9.1–12 min, 95% B. Samples were analyzed in randomized order with interspersed quality controls to ensure system stability. Mass spectrometric analysis was conducted using an Orbitrap Exploris^™^ 480 in both positive and negative ESI modes, with optimized parameters including sheath gas (50 arb), auxiliary gas (2 arb), ion source temperature (350 °C), and spray voltage (3500 V for positive mode; 2800 V for negative mode). Full-scan MS data were acquired from m/z 70–1200 with a resolution of 60,000, followed by data-dependent MS/MS analysis. Raw data were processed using ProteoWizard for format conversion and XCMS for peak alignment, retention time correction, and peak area extraction. Data preprocessing included null value filtering (> 50% missing values removed), KNN-based imputation, and removal of features with RSD > 50% in quality controls, ensuring robust metabolomic analysis.

#### Cell line culture

AML12 cell line was purchased from the Cell Bank of Type Culture Collection of the Chinese Academy of Sciences (Shanghai, China). AML12 cells were cultured in DMEM/F-12 medium (Gibco, C11330500BT, USA) supplemented with 5% fetal bovine serum (Vazyme, F101-01, China), and 1% penicillin–streptomycin. The cell lines were maintained in a humidified incubator at 37 °C with 5% CO2.

#### Cell transfection

AML12 cells were transfected with Nrf2 small interfering RNA (siRNA, 5′-CCGGCATTTCACTAAACACAA-3′, Haixing Biosciences, China) or a non-targeting control siRNA (siNC) using Lipofectamine 2000 (Invitrogen, USA) according to the manufacturer’s instructions. Briefly, cells were seeded in 6-well plates at 50–60% confluence and transfected with siRNA at a final concentration of 100 nM per well (2 mL medium) in Opti-MEM medium. After 8 h, the transfection mixture was replaced with complete DMEM/F12 medium containing 10% FBS. Cells were collected 48 h post-transfection for subsequent analyses.

#### Cell viability assay

The cell viability assay was performed using a Cell Counting Kit-8 (CCK-8, Yeasen, 40203ES80) according to the manufacturer’s instructions. Briefly, AML12 cells were seeded in 96-well plates at a density of 5 × 10^3^ cells per well and allowed to adhere. Following treatment, 10 μL of CCK-8 reagent was added to each well, and the plates were subsequently incubated at 37 °C for 2 h in a humidified atmosphere. The optical density (OD) at 450 nm was then measured using a microplate reader to quantify cell viability.

#### Cellular ROS detection

Intracellular ROS levels were detected using 2′,7′-dichlorodihydrofluorescein diacetate (DCFH-DA) probe (S0035S, Beyotime Biotechnology) following the established protocol. Cells were seeded in 6-well plates at a density of 1 × 10^5^ cells per well and allowed to adhere. The H2DCFDA stock solution was diluted with serum-free medium to achieve a final working concentration of 20 μM. After experimental treatments, cells were incubated with 1 mL of the working solution for 30 min at 37 °C in the dark. To remove excess probe and minimize background fluorescence, cells were washed twice with pre-warmed PBS. Fluorescence images were captured using a fluorescence microscope (Nikon, Japan) with consistent exposure settings across all samples.

#### Iron assay

Iron concentrations in cell supernatants were measured using a commercial Iron Assay Kit (Elabscience, E-BC-K773-M) following the manufacturer’s protocol.

For intracellular ferrous iron detection, AML12 cells were plated in confocal dishes and treated with experimental compounds. After triple washing with HBSS, cells were incubated with 1 μM FerroOrange probe (DojinDo, Japan) at 37 °C for 30 min. Fluorescence imaging was immediately performed using a confocal microscope (FV12-IXCOV, Olympus, Japan), and quantitative analysis was conducted with ImageJ 1.53c software.

Intracellular Fe^2^⁺ levels in AML12 cells were quantified using a Cell Ferrous Iron Colorimetric Assay Kit (Elabscience, E-BC-K881-M). After digestion and collection, cells were resuspended in buffer and lysed on ice for 10 min. The lysates were centrifuged at 15,000 × g for 10 min, and the clarified supernatants were obtained. A 80-µL aliquot of each sample was combined with the chromogenic reagent and incubated sequentially at 37 °C for 40 min and 10 min. Absorbance was subsequently determined at 593 nm using a Synergy H1 microplate reader (BioTek).

#### Transmission electron microscopy

Cell samples were fixed in 2.5% glutaraldehyde for 2 h at room temperature and then stored at 4 °C overnight. Subsequently, samples were post-fixed with 1% osmium tetroxide for 1 h, washed, dehydrated through an ethanol gradient (30, 50, 70 and 95%, 5 min per step), embedded and polymerized at 60 °C for 48 h. Ultrathin sections of 85 nm were cut and observed in a Tecnai 12 BioTwin transmission electron microscope (FEI Company, Eindhoven, The Netherlands) at 120 keV.

For liver tissue microscopy, the fixed tissues were embedded using EPON as previously descried. Ultrathin sections of 70 nm were cut using an ultramicrotome (Leica Microsystems, UC6) with a diamond knife (Diatome, Biel, Switzerland) and stained with 1.5% uranyl acetate at 37 °C for 15 min and lead citrate solution for 4 min. Electron micrographs were taken with a JEM-2100 Plus Transmission Electron Microscope (JEOL), equipped with Camera OneView 4 K 16 bit (Gatan) and software Digital Micrograph (Gatan).

For analysis, the length and morphology of each mitochondrion was determined in ImageJ following manual drawing of single organelle. All parameters obtained from one field of view were averaged together.

#### RNA isolation and quantitative real-time PCR (qRT-PCR)

Total RNA from the tissue or cells was extracted using TRIzol reagent (Life Technologies) according to the manufacturer’s protocol. Quantification was performed on a NanoDrop 8000 spectrophotometer (Thermo scientific), and 500 ng of total RNA per sample was reverse transcribed into complementary DNA using HiFiScript All-in-one RT Master Mix (CWBIO). Real-time PCR was performed using the SYBR qPCR Mix (CWBIO) according to the manufacturer’s instructions. Signals were detected using a CFX96 Real-time System (Bio-Rad). The 2^−△△CT^ approach was employed for determining mRNA relative transcription, with glyceraldehyde-3-phosphate dehydrogenase (GAPDH) being the loading reference. The primer sequences were listed in Table S1.

####  Western blot analysis

For Western blotting, cells or tissue were collected and lysed in radio immunoprecipitation assay (RIPA) buffer. After centrifugation at 15,000 g for 5 min at 4 C, we collected the supernatant as the protein lysate. Protein samples were separated by sodium dodecyl sulfate–polyacrylamide gel electrophoresis (SDS-PAGE) using 10% gel, transferred to a 0.45-μm pore-sized polyvinylidene difluoride (PVDF) membrane (Millipore). The membranes were blocked with 5% fat-free milk and then incubated with certain primary and secondary antibody. Eventually, the bands were visualized with enhanced chemiluminescence. The information on the primary and secondary antibodies used was listed in the Table S1.

#### Statistical analysis

The data were indicated by mean ± standard deviation (SD). GraphPad Prism 9.3.0 (GraphPad Software, USA) was employed for statistical analysis. All normally distributed data were analyzed using Student’s *t*-test or one-way ANOVA followed by Tukey’s post hoc analysis. p < 0.05 stood for a significant difference.

## Result

### Effects of GYJST on the HFD induced mice model in vivo

To investigate the therapeutic potential of GYJST against MAFLD, we established a disease model by feeding mice a HFD for 12 weeks, followed by an 8-week treatment period with low-, medium-, and high-dose GYJST (Fig. 1A). In addition, Feno was considered as a positive group. Notably, while HFD-fed mice exhibited significant weight gain, GYJST treatment demonstrated a dose-dependent reduction in body weight, with the most pronounced effect observed in the GYJST-H group (Fig. 1B). More interestingly, GYJST could reduce liver weight and liver index in MAFLD mice (Fig. 1C, D). Subsequent biochemical analyses revealed that serum TC, TG, AST, and ALT levels were markedly elevated in the model group compared to the control group. Following treatment, GYJST unquestionably decreased serum TC, TG, AST, and ALT (Fig. 1E, F). These findings strongly suggest that GYJST effectively alleviates liver steatosis and improves liver function in the HFD induced mouse model.

Quantitative assessment using the NAS score (Fig. 1G) demonstrated a dose-dependent reduction in hepatic steatosis, inflammation, and ballooning in GYJST-treated groups compared to the model group. Macroscopic analysis (Fig. 1H) revealed pronounced hepatomegaly and yellowish discoloration in model mice, both of which were markedly alleviated following GYJST treatment, with the most significant improvement observed in the GYJST-H group. Histological evaluation through H&E staining (Fig. 1L) revealed extensive macrovesicular steatosis, ballooning degeneration, and lobular disarray in the livers of model mice. In contrast, GYJST treatment mitigated these pathological changes, showing a dose-dependent decrease in lipid accumulation and restoration of normal hepatic architecture. Additionally, Masson’s trichrome (Fig. 1I, L), Sirius Red (Fig. 1J, L), Oil Red O (Fig. 1K, L), further corroborated the protective effects of GYJST against hepatic fibrosis, lipid deposition respectively. Quantitative analysis of the positive staining areas (Fig. 1I, K) indicated that GYJST significantly reduced the percentage of fibrotic areas (Masson and Sirius Red), and lipid-laden regions (Oil Red O) in a dose-dependent manner, with the GYJST-H group showing the most pronounced effects. These comprehensive findings collectively demonstrate the therapeutic efficacy of GYJST in ameliorating MAFLD progression through multiple mechanisms. Given that this study is the first to explore the therapeutic efficacy of GYJST on MAFLD, the high-dose group was chosen for a comprehensive multi-omics analysis and in vivo validation.

### Component characterization and network pharmacological analysis

#### Identification of the compounds in GYJST by UPLC-HRMS

**Fig. 2:**
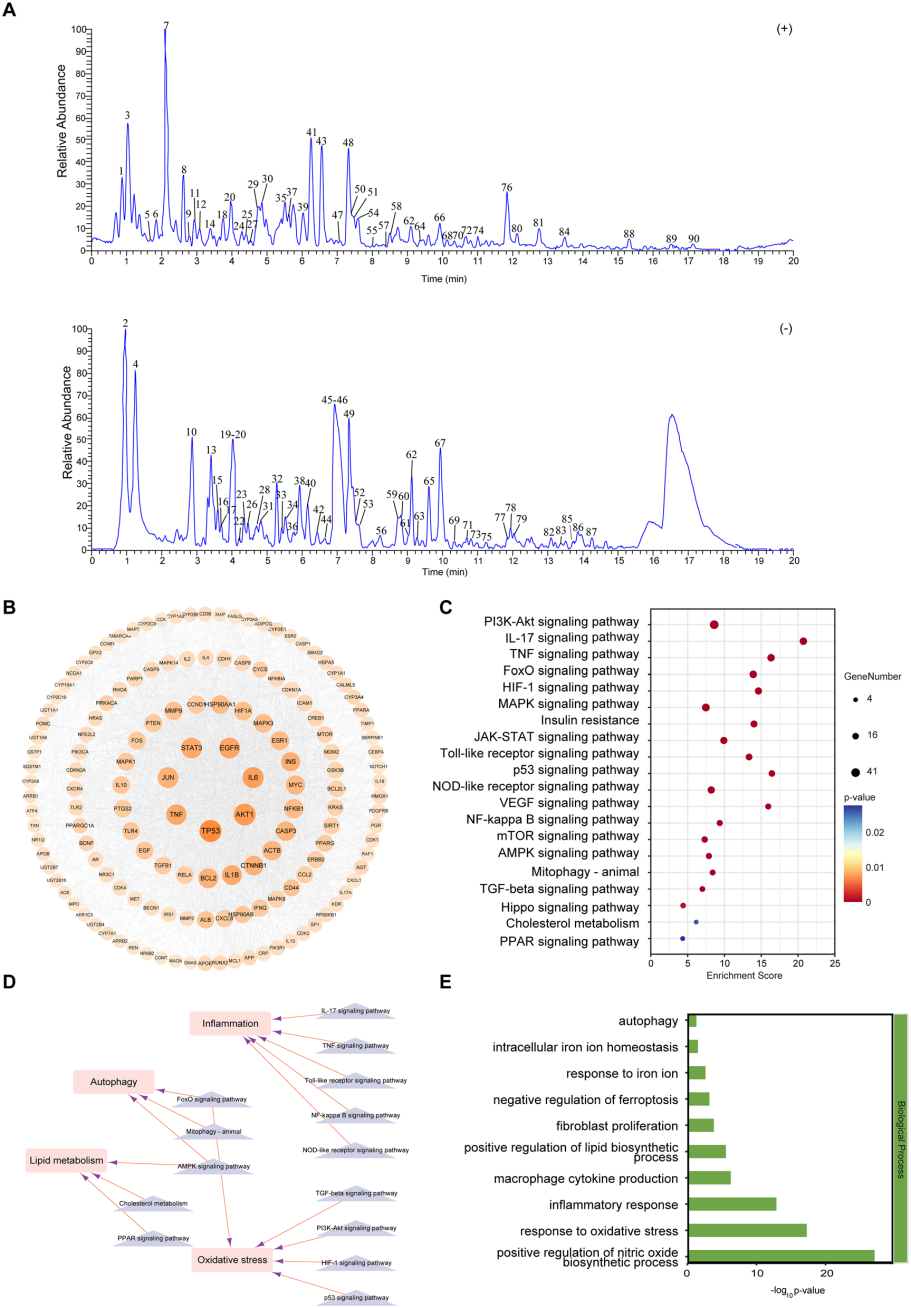
Network analysis of GYJST treating MAFLD. **A** 90 main compounds from GYJST were identified by UPLC-HRMS. **B** Acquirement of the 148 core targets with a sensitivity at least twice the median. **C**, **D** KEGG pathway enrichment analysis and characterization of pathway attributes. **E** GO enrichment analysis of core targets in biological processes

UPLC-HRMS was utilized to establish the quality control of GYJST and to identify the compounds of GYJST. The typical base peak and ion flow chromatographs are shown in Fig. 2A. Based on databases and literature, a total of 90 main compounds from GYJST were tentatively characterized (Fig. 2A). The classes of compounds mainly included dipeptides, aminoacids, flavonols, pyridine alkaloids, simple phenolic acids, simple coumarins, cinnamic acids and derivatives, flavones, dicarboxylic acids, simple indole alkaloids, tripeptides, flavanones, pregnane steroids, Phenylalanine derived alkaloids. Detailed characterizations for each compound are provided in Table 1.

**Table 1:** Characterization of compounds of YQHX by UPLC-HRMS

No.	RT min	m/z	Formula	ppm	Compound name	Adduct	Score	PubCHEM	CAS	Class	Product Ion
1	0.89	377.1055	C_19_H_21_CN_2_O_2_S	8.2	Capsazepine	[M + H]^+^	0.9911	2,733,484	138,977–28-3	Amarylidaceae alkaloids	[377.11, 377.19, 118.09, 215.05, 104.11]
2	0.97	109.0284	C_6_H_6_O_2_	10	Pyrocatechol	[M-H]^−^	0.9873	289	120–80-9	Simple phenolic acids	[109.03, 108.02, 91.02, 110.03, 109.01]
3	1.04	321.1157	C_20_H_16_O_4_	11.3	Corylin	[M + H]^+^	0.9999	5,316,097	53,947–92-5	Isoflavones	[321.12, 321.18, 159.06, 321.21, 322.12]
4	1.22	222.9914	C_11_H_8_O_4_S_2_	9	5-(Phenylsulfonyl)-2-	[M-H-CO2]^−^	0.9712	220,257	5324–78-7	Others	[222.99, 96.96, 143.03, 115.04, 113.02]
Thiophenecarboxylic acid											
5	1.71	166.1227	C_10_H_15_NO	0.7	Hordenine	[M + H]^+^	0.9971	68,313	539–15-1	Amarylidaceae alkaloids	[121.06, 93.07, 103.05, 91.05, 166.12]
6	1.81	270.0958	C_13_H_22_N_4_O_3_S	17.7	Ranitidine	[M + H-C_2_H7N]^+^	0.9302	3,001,055	66,357–35-5	Others	[270.1, 270.13, 180.06, 108.04, 252.09]
7	2.10	226.0689	C_10_H_11_NO_5_	10.1	Madurastatin B2	[M + H]^+^	0.9835	11,368,103	768,384–52-7	Dipeptides	[226.07, 226.11, 110.06, 208.1, 180.07]
8	2.61	231.0654	C_13_H_10_O_4_	0.2	Methanone	[M + H]^+^	0.9963	77,093	3555–86-0	Acyl phloroglucinols	[153.02, 105.03, 231.07, 95.05, 97.03]
9	2.70	263.0551	C_13_H_10_O_6_	0.2	Macurin	[M + H]^+^	0.9929	68,213	519–34-6	Acyl phloroglucinols	[153.02, 263.14, 263.06, 137.02, 177.11]
10	2.88	153.0184	C_7_H_6_O_4_	6.2	Pyrocatechuic acid	[M-H]^−^	0.9987	19	303–38-8	Simple phenolic acids	[109.03, 153.02, 108.02, 110.03, 91.02]
11	2.99	159.0917	C_18_H_15_C_l_N_2_O_3_	0.5	Benzotript	[M + H-C_8_H_5_ClO_3_]^+^	0.9934	2,787,526	39,544–74-6	Dipeptides	[132.08, 159.09, 130.07, 142.07, 117.06]
12	3.10	227.0816	C_13_H_11_C_l_N_2_O_2_	0.2	3-Pyridinecarboxamide	[M + H-HCl]^+^	0.9917	840,301	56,149–30-5	Pyridine alkaloids	[227.08, 212.06, 184.06, 199.09, 197.07]
13	3.41	191.0554	C_7_H_12_O_6_	4.1	Quinate	[M-H]^−^	0.9975	6508	80–75-1	Simple phenolic acids	[191.06, 93.03, 127.04, 192.06, 111.01]
14	3.42	342.1699	C_20_H_24_NO_4_	0.4	Phellodendrine	[M]^+^	0.999	59,818	104,112–82-5	Isoquinoline alkaloids	[192.1, 177.08, 342.17, 149.08, 306.13]
15	3.52	367.1031	C_17_H_20_O_9_	1.4	5-Feruloylquinic acid	[M-H]^−^	0.989	10,133,609	40,242–06-6	Cinnamic acids and derivatives	[193.05, 134.04, 117.03, 149.06, 367.1]
16	3.68	153.0183	C_7_H_6_O_4_	5	Gentisic acid	[M-H]^−^	0.9843	3469	490–79-9	Simple phenolic acids	[109.03, 108.02, 153.02, 110.03, 109.06]
17	3.70	633.0737	C_27_H_22_O_18_	1	Corilagin	[M-H]^−^	0.9834	73,568	23,094–69-1	Gallotannins	[301.0, 633.07, 275.02, 229.01, 257.01]
18	3.76	144.0809	C_19_H_26_N_2_	0.3	Curan	[M + H-C_9_H_17_N]^+^	0.9923	9,548,844	34,174–79-3	Corynanthe type	[144.08, 143.07, 115.05, 128.05, 103.05]
19	3.99	179.0343	C_9_H_8_O_4_	3.7	Caffeic acid	[M-H]^−^	0.9979	2518	331–39-5	Cinnamic acids and derivatives	[135.04, 179.03, 134.04, 136.05, 107.05]
20	4.01	135.1172	C_10_H_16_O	1.7	Carveol	[M + H-H2O]^+^	0.9684	7438	99–48-9	Menthane monoterpenoids	[91.05, 107.09, 105.07, 93.07, 135.12]
21	4.01	163.0391	C_9_H_8_O_3_	5.3	4-Hydroxycinnamic acid	[M-H]^−^	0.9987	637,542	501–98-4	Cinnamic acids and derivatives	[119.05, 163.04, 120.05, 93.03, 91.05]
22	4.19	421.1715	C_25_H_26_O_6_	13.5	5-Hydroxyorientanol F	[M-H]^−^	0.9112	11,964,495	927,898–04-2	Isoflavanones	[259.12, 421.17, 183.1, 139.11, 111.01]
23	4.29	183.0292	C_8_H_8_O_5_	4.1	Benzoic acid	[M-H]^−^	0.9912	78,016	4319–02-2	Cinnamic acids and derivatives	[168.01, 124.02, 183.03, 169.01, 139.0]
24	4.31	403.148	C_21_H_22_O_8_	22.8	Hexamethylquercetagetin	[M + H]^+^	0.9973	386,331	1251–84-9	Flavonols	[403.15, 385.14, 341.15, 375.15, 100.78]
25	4.40	123.0443	C_13_H_16_O_7_	1.5	Helicid	[M + H-C_6_H_10_O_5_]^+^	0.9907	12,896,796	80,154–34-3	Simple phenolic acids	[95.05, 123.06, 96.04, 105.04, 123.04]
26	4.44	335.0772	C_16_H_18_O_9_	0.1	Cryptochlorogenic acid	[M-H-H_2_O]^−^	0.9638	9,798,666	905–99-7	Cinnamic acids and derivatives	[161.02, 335.08, 133.03, 93.03, 135.04]
27	4.54	501.1604	C_22_H_28_O_13_	0.8	Haploperoside c	[M + H]^+^	0.9996	20,106,091	97,938–28-8	Simple coumarins	[193.05, 151.04, 125.06, 129.05, 147.04]
28	4.71	609.146	C_27_H_30_O_16_	0.8	Rutin	[M-H]^−^	0.9787	5,280,805	153–18-4	Flavonols	[300.03, 271.02, 609.15, 301.04, 255.03]
29	4.73	611.1613	C_27_H_31_O_16_	1.9	Tulipanin	[M]^+^	0.9958	5,492,231	15,674–58-5	Anthocyanidins	[303.05, 229.05, 153.02, 257.04, 137.02]
30	4.80	347.2041	C_23_H_26_N_2_O	22.1	Roxindole	[M + H]^+^	0.9993	219,050	112,192–04-8	Simple oxindole alkaloids	[347.2, 348.21, 347.3, 347.28, 347.11]
31	4.86	463.088	C_21_H_20_O_12_	0.4	hyperoside	[M-H]^−^	0.9481	5,280,804	482–35-9	Flavonols	[300.03, 271.02, 463.09, 301.03, 255.03]
32	5.30	579.1716	C_27_H_32_O_14_	0.1	Naringin	[M-H]^−^	0.9627	442,428	10,236–47-2	Flavanones	[151.0, 271.06, 119.05, 579.17, 107.01]
33	5.43	433.0773	C_20_H_18_O_11_	1	Reynoutrin	[M-H]^−^	0.9463	5,320,861	549–32-6	Flavonols	[300.03, 301.03, 271.02, 255.03, 433.08]
34	5.51	515.119	C_25_H_24_O_12_	0.6	Isochlorogenic acid C	[M-H]^−^	0.9701	6,474,309	57,378–72-0	Cinnamic acids and derivatives	[173.04, 353.09, 135.04, 179.03, 191.06]
35	5.55	449.1079	C_21_H_20_O_11_	1.4	Quercetin 7-rhamnoside	[M + H]^+^	0.9963	5,748,601	22,007–72-3	Flavonols	[287.06, 303.05, 153.02, 229.05, 137.02]
36	5.71	115.0754	C_6_H_12_O_2_	8.4	Caproic acid	[M-H]^−^	0.9636	8892	142–62-1	Branched fatty acids	[115.08, 115.92, 99.92, 113.1, 100.26]
37	5.72	340.1543	C_20_H_21_NO_4_	1.3	Canadine	[M + H]^+^	0.9936	34,458	522–97-4	Isoquinoline alkaloids	[176.07, 340.15, 149.06, 119.05, 91.05]
38	5.88	161.0447	C_12_H_22_O_11_	5.7	3alpha-Mannobiose	[M-2H-H_2_O]^2−^	0.9156	11,013,287	23,745–85-9	Disaccharides	[161.06, 131.03, 119.05, 161.04, 113.02]
39	6.12	419.0974	C_20_H_18_O_10_	0.2	Kaempferol 3-xyloside	[M + H]^+^	0.9972	21,310,440	61,117–16-6	Flavonols	[287.05, 153.02, 121.03, 165.02, 213.05]
40	6.16	235.0607	C_18_H_22_O_10_	2.8	Picraquassioside A	[M-H-C_6_H_10_O_5_]^−^	0.9646	10,250,244	169,312–28-1	Triterpenoid saponins	[235.06, 177.02, 217.05, 236.06, 219.03]
41	6.32	447.0924	C_21_H_18_O_11_	2.4	Baicalin	[M + H]^+^	0.9997	64,982	21,967–41-9	Flavones	[271.06, 123.01, 253.05, 169.01, 103.05]
42	6.41	173.1176	C_9_H_18_O_3_	3.8	9-Hydroxynonanoic acid	[M-H]^−^	0.9959	138,052	3788–56-5	Hydroxy fatty acids	[173.12, 127.11, 174.12, 93.03, 111.01]
43	6.54	366.17	C_22_H_24_NO_4_	0.3	Dehydrocorydaline	[M +]^+^	0.9969	34,781	30,045–16-0	Isoquinoline alkaloids	[366.17, 350.14, 322.14, 351.15, 308.13]
44	6.57	187.0967	C_9_H_16_O_4_	2.8	Azelaic acid	[M-H]^−^	0.9733	2266	123–99-9	Dicarboxylic acids	[125.1, 187.1, 169.09, 97.06, 123.08]
45	6.99	267.16	C_15_H_24_O_4_	1.3	Acoric acid	[M-H]^−^	0.9957	15,558,301	5956–06-9	Acorane sesquiterpenoids	[267.16, 267.13, 96.96, 223.13, 204.13]
46	7.04	287.056	C_15_H_12_O_6_	1	Eriodictyol	[M-H]^−^	0.9943	440,735	552–58-9	Flavanones	[135.04, 151.0, 107.01, 287.06, 125.02]
47	7.08	285.0757	C_16_H_12_O_5_	0.4	Calycosin	[M + H]^+^	0.9953	5,280,448	20,575–57-9	Isoflavones	[285.08, 270.05, 253.05, 225.05, 213.05]
48	7.32	439.16	C_21_H_26_O_10_	0.3	sec-o-Glucosylhamaudol	[M + H]^+^	0.9951	10,478,277	80,681–44-3	Chromones	[277.11, 259.1, 205.05, 217.05, 439.23]
49	7.33	301.0351	C_15_H_10_O_7_	1.6	Morin	[M-H]^−^	0.9887	5,281,670	480–16-0	Flavonols	[301.04, 151.0, 179.0, 107.01, 121.03]
50	7.42	287.0549	C_15_H_10_O_6_	0.4	Luteolin	[M + H]^+^	0.9997	5,280,445	491–70-3	Flavones	[287.05, 153.02, 135.04, 241.05, 137.02]
51	7.44	301.0707	C_16_H_12_O_6_	1	Sydowinin A	[M + H]^+^	0.9865	23,872,092	58,450–01-4	Methyl xanthones	[301.07, 269.04, 286.05, 167.03, 301.1]
52	7.46	191.0343	C_26_H_32_O_11_	4.1	Camaldulenside	[M-H-C_16_H_24_O_7_]^−^	0.9982	10,625,791	327,022–93-5	Chromones	[191.03, 192.04, 147.04, 123.04, 103.05]
53	7.54	151.0392	C_8_H_8_O_3_	5.8	2,4-Cresotic acid	[M-H]^−^	0.998	5788	50–85-1	Simple phenolic acids	[107.05, 151.04, 101.02, 108.05, 151.06]
54	7.57	200.0707	C_12_H_11_NO_3_	0.3	N-(2-Furanylmethyl)-	[M + H-H_2_O]^+^	0.9968	332,769	501,661–50-3	Others	[200.07, 172.08, 145.06, 144.08, 199.06]
					anthranilic acid						
55	8.00	187.0391	C_11_H_6_O_3_	0.3	Ficusin	[M + H]^+^	0.9585	6199	66–97-7	Simple coumarins	[187.04, 131.05, 115.05, 143.05, 159.04]
56	8.26	271.061	C_15_H_12_O_5_	0.7	Naringenin	[M-H]^−^	0.9804	439,246	480–41-1	Flavanones	[271.06, 119.05, 151.0, 107.01, 93.03]
57	8.51	310.1049	C_18_H_17_NO_5_	8.3	Tranilast	[M + H-H_2_O]^+^	0.9703	5,282,230	53,902–12-8	Cinnamic acid amides	[310.11, 121.07, 93.07, 310.14, 190.09]
58	8.69	275.055	C_14_H_10_O_6_	0.5	Simonyellin	[M + H]^+^	0.9961	138,756,224	173,322–91-3	Isocoumarins	[275.05, 260.03, 232.04, 176.05, 187.04]
59	8.72	313.0352	C_16_H_10_O_7_	0.5	Endocrocin	[M-H]^−^	0.9816	160,483	481–70-9	Anthraquinones and anthrones	[269.05, 313.04, 225.06, 241.05, 197.06]
60	8.8	269.0818	C_16_H_14_O_4_	0.6	Alpinetin	[M-H]^−^	0.9501	154,279	36,052–37-6	Flavanones	[269.08, 269.05, 165.02, 254.06, 227.07]
61	8.98	371.1134	C_20_H_20_O_7_	0.6	Averantin	[M-H]^−^	0.9447	10,044,783	5803–62-3	Anthraquinones and anthrones	[371.11, 272.03, 271.02, 370.97, 258.02]
62	9.10	271.0601	C_15_H_10_O_5_	0.3	Baicalein	[M + H]^+^	0.9988	5,281,605	491–67-8	Flavones	[271.06, 123.01, 253.05, 103.05, 169.01]
63	9.23	315.051	C_16_H_12_O_7_	0.4	Pinoquercetin	[M-H]^−^	0.9441	5,281,679	491–49-6	Flavonols	[315.05, 165.02, 121.03, 193.01, 109.03]
64	9.52	213.0547	C_14_H_8_O_4_	0.1	Alizarin	[M + H–CO]^+^	0.9981	6293	72–48-0	Anthraquinones and anthrones	[213.05, 185.06, 157.06, 139.05, 129.07]
65	9.61	227.1283	C_12_H_20_O_4_	2.6	Traumatic acid	[M-H]^−^	0.9959	5,283,028	6402–36-4	Dicarboxylic acids	[183.14, 165.13, 227.13, 184.14, 209.12]
66	9.92	221.0809	C_12_H_12_O_4_	10.2	Eugenitin	[M + H]^+^	0.9806	3,083,581	480–12-6	Chromones	[221.08, 206.06, 205.05, 179.03, 221.03]
67	9.98	315.051	C_16_H_12_O_7_	0.1	Rhamnetin	[M-H]^−^	0.9647	5,281,691	90–19-7	Flavonols	[315.05, 165.02, 121.03, 300.03, 97.03]
68	10.13	313.2008	C_18_H_24_N_4_O	11.8	Granisetron	[M + H]^+^	0.9949	5,284,566	109,889–09-0	Tropane alkaloids	[313.2, 313.16, 206.64, 313.04, 313.23]
69	10.37	192.1025	C_11_H_15_NO_2_	3.6	4-Phenylisovaline	[M-H]^−^	0.9928	232,323	5472–95-7	Aminoacids	[192.1, 93.02, 135.03, 193.11, 148.04]
70	10.41	250.1767	C_15_H_23_NO_2_	8.4	Alprenolol	[M + H]^+^	0.9998	2119	13,655–52-2	Phenylalanine-derived alkaloids	[250.18, 208.8, 251.18, 159.79]
71	10.70	283.061	C_16_H_12_O_5_	0.4	Wogonin	[M-H]^−^	0.9979	5,281,703	632–85-9	Flavones	[268.04, 283.06, 163.0, 110.0, 165.99]
72	10.71	531.1862	C_27_H_30_O_11_	0.9	Icariin I	[M + H]^+^	0.9987	5,745,470	56,725–99-6	Flavonols	[313.07, 369.13, 135.04, 243.07, 531.29]
73	10.97	498.2894	C_26_H_45_NO_6_S	0.5	Taurodeoxycholic acid	[M-H]^−^	0.9976	2,733,768	516–50-7	Cholane steroids	[498.29, 106.98, 124.01, 203.59, 124.57]
74	11.00	294.1677	C_17_H_19_N_5_	12.1	Anastrozole	[M + H]^+^	0.9947	2187	120,511–73-1	Others	[294.17, 276.23, 294.24, 122.06, 123.07]
75	11.21	253.1444	C_14_H_22_O_4_	1.1	3-Furancarboxylic acid	[M-H]^−^	0.9649	9,881,506	1,234,694–20-2	Paraconic acids and derivatives	[253.14, 209.15, 191.14, 109.06, 253.17]
76	11.81	447.2516	C_24_H_40_O_5_	24.3	Hyocholic acid	[M + K]^+^	0.8104	139,291,826	547–75-1	Cholane steroids	[447.25, 465.25, 411.22, 429.23, 393.21]
77	11.82	407.28	C_24_H_40_O_5_	0.4	Cholic acid	[M-H]^−^	0.9979	221,493	81–25-4	Cholane steroids	[407.28, 343.26, 289.22, 345.28, 363.29]
78	11.97	269.0817	C_16_H_14_O_4_	0.5	Cardamonin	[M-H]^−^	0.9769	641,785	19,309–14-9	Chalcones	[269.08, 226.06, 254.06, 122.0, 165.02]
79	12.08	351.0871	C_20_H_16_O_6_	0.7	Parvisoflavone B	[M-H]^−^	0.9378	14,550,385	50,277–02-6	Isoflavones	[351.09, 133.03, 217.05, 335.06, 336.06]
80	12.16	657.3622	C_35_H_54_O_10_	2.3	Dianthosaponin C	[M + Na]^+^	0.9788	56,776,384	1,370,510–85-2	Oleanane triterpenoids	[657.36, 569.31, 639.35, 587.32, 585.3]
81	12.74	424.3426	C_25_H_45_NO_4_	1	Linoleoylcarnitine	[M + H]^+^	0.9902	6,450,015	36,816–10-1	Fatty acyl carnitines	[424.34, 144.1, 95.09, 97.1, 109.1]
82	13.04	359.1862	C_21_H_28_O_5_	0.8	Aldosterone	[M-H]^−^	0.9516	5839	52–39-1	Pregnane steroids	[359.19, 125.06, 189.13, 359.28, 205.05]
83	13.33	367.1184	C_21_H_20_O_6_	0.6	Icaritin	[M-H]^−^	0.9891	5,318,980	118,525–40-9	Flavonols	[367.12, 309.04, 297.04, 281.05, 175.0]
84	13.44	263.1431	C_19_H_18_O	0.3	Alnustone	[M + H]^+^	0.9899	5,317,598	33,457–62-4	Linear diarylheptanoids	[91.05, 105.07, 95.09, 263.24, 263.14]
85	13.70	265.1477	C_12_H_26_O_4_S	0.8	Laurylsulfuric acid	[M-H]^−^	0.9413	8778	151–41-7	Fatty alcohols	[96.96, 265.15, 96.97, 265.22, 95.95]
86	13.92	531.3323	C_31_H_48_O_7_	1.2	Phytolaccagenin	[M-H]^−^	0.9676	21,594,228	1802–12-6	Ursane and Taraxastane triterpenoids	[531.34, 125.06, 111.04, 256.24, 301.14]
87	14.23	441.228	C_26_H_34_O_6_	1.7	Rhodomyrtone	[M-H]^−^	0.9779	44,237,956	468,757–69-9	Dimeric phloroglucinols	[441.23, 111.04, 303.2, 259.21, 232.07]
88	15.37	443.3138	C_45_H_72_O_12_	4	Oligomycin B	[M + H-C_17_H_30_O_8_]^+^	0.9895	5,281,900	11,050–94-5	Others	[443.32, 188.07, 425.3, 159.09, 146.06]
89	16.51	537.3939	C_35_H_52_O_4_	0.3	Hyperforin	[M + H]^+^	0.9386	441,298	11,079–53-1	Acyl phloroglucinols	[203.07, 277.14, 221.08, 413.27, 219.1]
90	17.15	505.092	C_30_H_16_O_8_	3.7	Hypericin	[M + H]^+^	0.9894	3663	548–04-9	Anthraquinones and anthrones	[505.09, 463.08, 407.09, 435.09, 392.07]

#### Target prediction of potential compounds of GYSJT against HFD induced mice

A total of 679 known target genes of the main compounds in GYJST were obtained from TCMSP, Swiss Target Prediction, CTD, ETCM and PharMapper databases after removing duplicates and transforming the protein names to gene symbols (Table S2).

Moreover, MAFLD-related targets were identified from the GeneCards, TTD and CTD, and OMIM databases, which together yielded a total of 38,694 genes (Table S3). The keywords used for the search were “non-alcoholic fatty liver disease” and “metabolic associated fatty liver disease”. Thus, a total of 665 intersected targets were obtained from GYJST and MAFLD related targets, which were selected as the foundational effector genes in subsequent analysis (Table S4).

To further assess the significance of potential targets, 665 common targets were uploaded to the STRING 11.0 database for analysis. Based on strict screening criteria (combined score > 0.7), 589 targets were subjected to network topological analysis, also known as protein–protein interaction analysis, leading to the construction of an interaction network. This network comprised 589 nodes and 5456 edges, with an average node degree of 12. Through network topological analysis and following the screening principle (targets with a sensitivity at least twice the median), 148 key targets were identified (Fig. 2B, Table S5).

To clarify the potential functions of these 148 key targets, gene annotation and functional enrichment analysis were performed via using the DAVID platform. The enrichment of KEGG signaling pathways demonstrated that GYJST treatment of liver mainly involved the inflammation, autophagy, lipid metabolism and oxidative stresss (Fig. 2C, D). More interestingly, the GO biological processes were involved with several main aspects of treating MAFLD, including alleviation of oxidative stress, inflammatory response, negative regulation of ferroptosis and autophagy (Fig. 2E). In addition, the GO molecular functions were associated with signaling receptor binding, iron ion binding, fibroblast growth factor receptor activity (Fig. S1A). Additionally, the GO cellular components were chromatin, mitochondrion, mitochondrial outer membrane (Fig. S1B).

#### GYJST regulated the expression of several genes in the liver

**Fig. 3:**
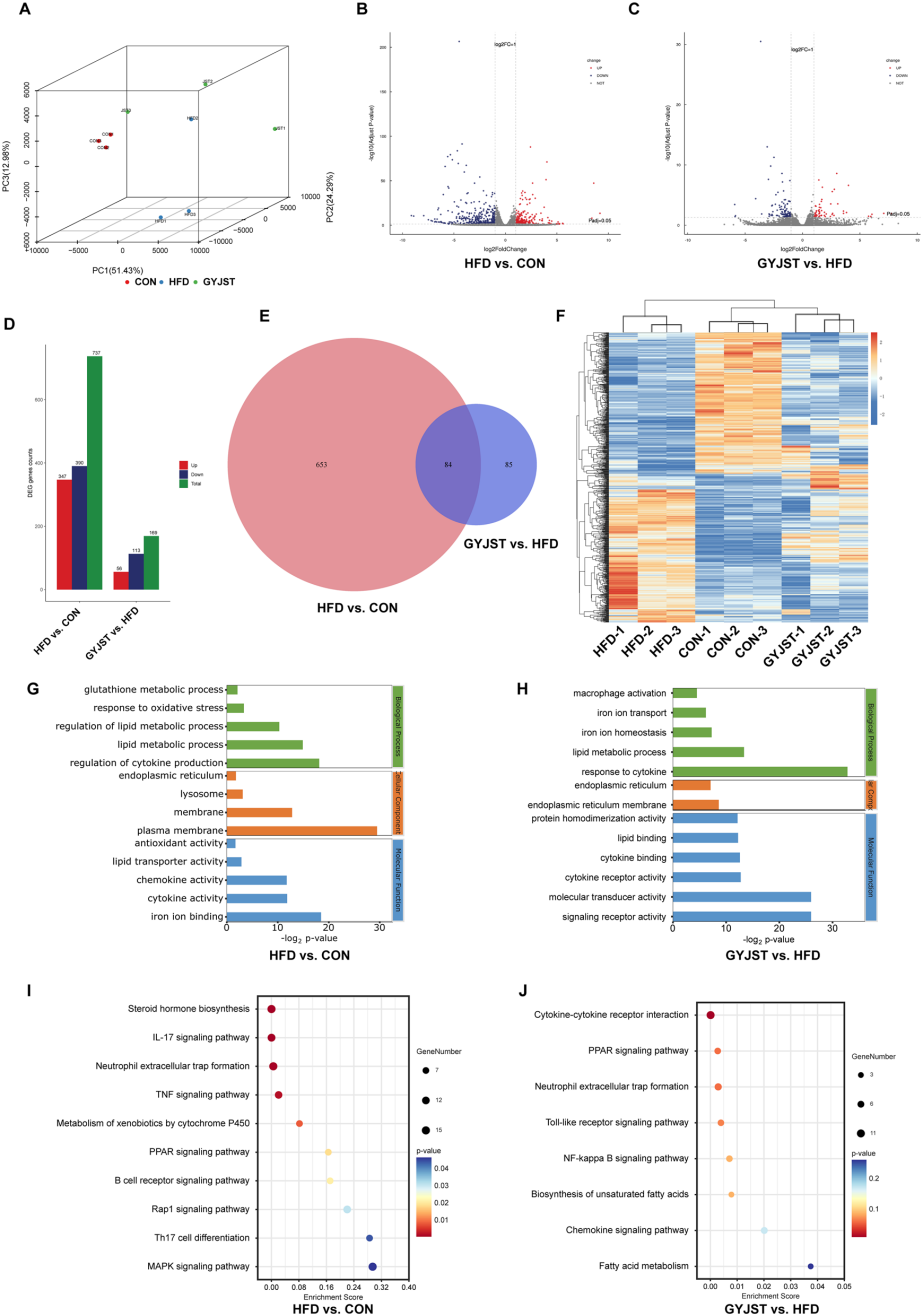
Effects of GYJST on gene expression in the liver of HFD-induced MAFLD. **A** Principal component analysis. **B** Volcano plots of gene expression differences in HFD versus Control. **C** Volcano plots of gene expression differences in GYJST versus HFD. **D** The up- and downregulated DEGs by the pairwise comparison of HFD versus Control and GYJST versus HFD. **E** Venn diagram of the DEGs among the three groups. **F** Heatmap showing the cluster analysis of DEGs. **G**, **H** GO analysis of differentially expressed genes in the HFD versus Control group and GYJST versus HFD group. **I**, **J** KEGG analysis of differentially expressed genes in the HFD versus Control groups and GYJST versus HFD groups

To gain a systematic understanding of the changes in the transcriptome of the liver, transcriptome sequencing of liver tissues from mice treated with or without GYJST was performed. The principal component analysis (PCA) results showed a clear distinction between the CON, HFD, and GYJST groups, reflecting the significant effects of GYJST treatment on liver gene expression (Fig. 3A). Through the analysis of differentially expressed genes (DEGs) (p < 0.05, |log2FC|> 1), 906 DEGs were obtained in the HFD versus CON and GYJST versus HFD groups (Fig. 3B, C). There were 737 DEGs identified between HFD and CON groups, with 347 up-regulated and 390 down-regulated. After the treatment with GYJST, a total of 169 DEGs were determined between GYJST and Mod groups, with 56 were up-regulated and 113 were down-regulated (Fig. 3D). By intersection analysis, there were 84 overlap DEGs between HFD versus CON and GYJST versus HFD groups (Fig. 3E), GYJST could restore 49.7% gene expression in HFD group to normal (Fig. 3F).

In the comparison between the HFD versus CON (Fig. 3G) and GYJST versus HFD (Fig. 3H) groups, the GO enrichment analysis primarily highlights processes such as glutathione metabolism, response to oxidative stress, lipid metabolism, and iron ion homeostasis. The pathway enrichment analysis of differential genes can effectively identify major pathways involved in HFD processes and GYJST treatment. Based on KEGG databases, pathway enrichment analysis for DEGs in HFD versus CON and GYJST versus HFD group were performed, respectively. It was shown that tumor necrosis factor (TNF) signaling pathway, interleukin-17 (IL-17) signaling pathway and peroxisome proliferators activated receptor (PPAR) signaling pathways were enriched with DEGs of both groups (Fig. 3I, J).

Furthermore, gene set enrichment analysis (GSEA) revealed that the PPAR signaling pathway was upregulated in the HFD group (Fig. S2A), which was reversed by GYJST treatment (Fig. S2B). In the HFD group, protein–protein interaction (PPI) network analysis revealed that prostaglandin-endoperoxide synthase 2 (COX2, also known as PTGS2) was significantly observed and interacted with glutathione S-transferase M2 (GSTM2), being considered a marker of ferroptosis and inflammation (Fig. S2C). More interestingly, COX2 and PPAR were found to be connected in the PPI network, especially in the GYJST treatment group (Fig. S2D). In addition, based on the annotation information from the Pfam database and the transcription factor family information from the DBD (Transcription Factor Prediction Database), transcription factor annotations were performed on the differentially expressed genes. The results are shown in Figs. S2E and S2F.

#### GYJST altered the proteomics profile

**Fig. 4:**
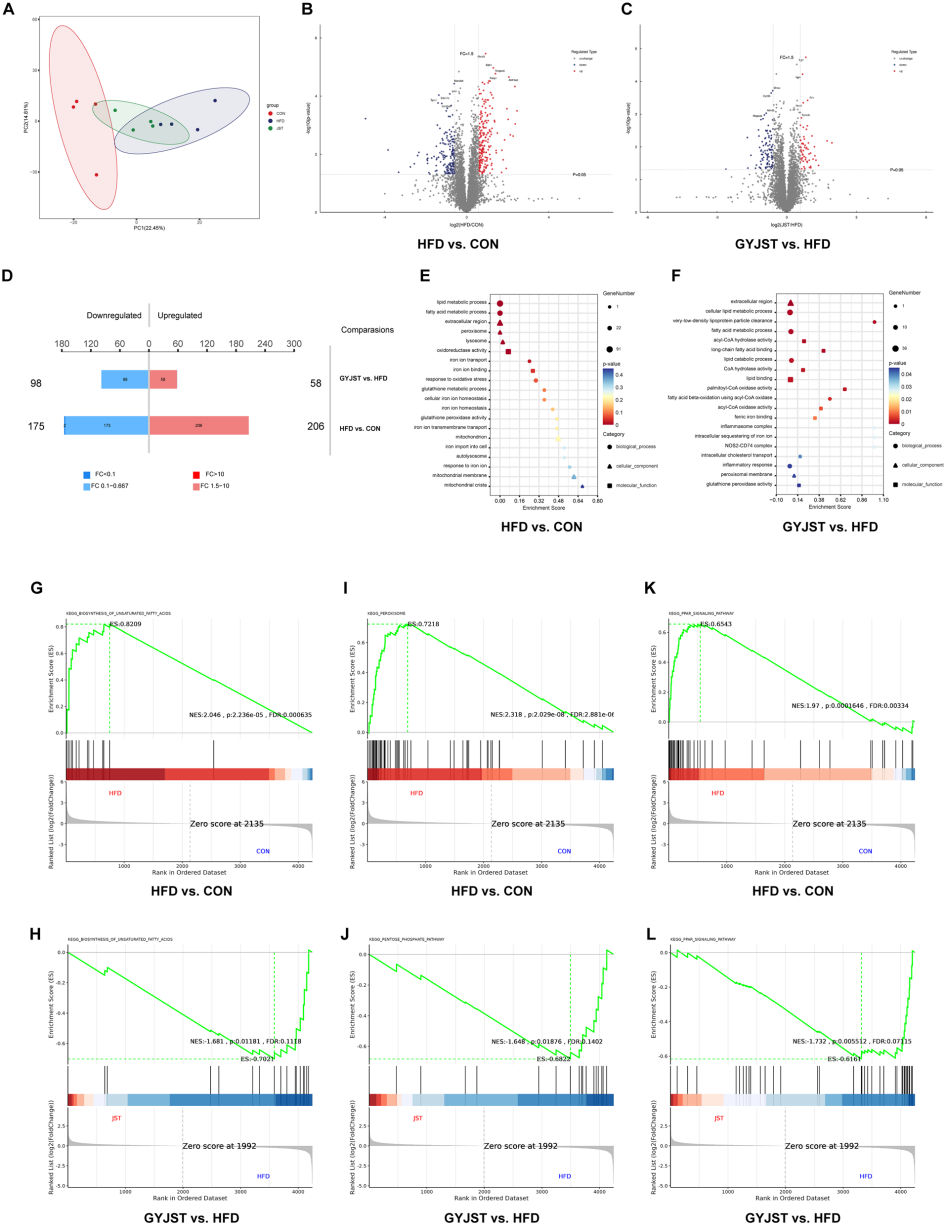
Effects of GYJST on protein expression in the liver of HFD-induced MAFLD. **A** Principal component analysis; **B** Volcano plots of protein expression differences in HFD versus Control; **C** Volcano plots of protein expression differences in GYJST versus HFD. **D** The up- and downregulated DEPs by the pairwise comparison of HFD versus Control and GYJST versus HFD. **E**, **F** GO analysis of differentially expressed proteins in the HFD versus Control groups and GYJST versus HFD groups. **G**–**L** GSEA for the biosynthesis of unsaturated fatty acids, peroxisome and PPAR signaling pathways in HFD versus CON and GYJST versus HFD

Proteomics is a powerful tool for the identification of novel biomarkers and potential protein targets. To reveal the proteomic characteristics of GYJST, the data independent acquisition (DIA) method was utilized to analyze proteomic changes in the bone marrow cell samples. The PCA results showed a clear distinction between CON, HFD and GYJST groups (Fig. 4A). In total, 537 differentially expressed proteins (DEPs) presented between the three groups (p < 0.05, FC > 1.5 or < 0.67) (Fig. 4B, C). There were 381 DEPs identified between HFD and CON groups, with 206 up-regulated and 175 down-regulated. After the treatment with GYJST, a total of 156. DEPs were determined between GYJST and HFD groups, with 58 up-regulated and 98 down-regulated (Fig. 4D).

Domains are generally the basis for interactions between proteins (or other tiny molecules). Key protein functions may alter as a result of variations within a domain or changes in amino acids. Consequently, the domains of proteins with differential expression were predicted using the domain prediction program Interproscan (Fig. S3A, B). The domain enrichment analysis before and after treatment revealed notable changes, suggesting potential mechanisms of therapeutic action. Metabolism-related domains, such as Thiolas domains and Short-chain dehydrogenase, showed decreased enrichment after treatment, indicating a partial alleviation of metabolic disorders. Iron metabolism and immune-related domains, including Ferritin-like domain and Immunoglobulin domains, remained enriched, suggesting that ferroptosis and immune responses continued to play a role post-treatment, albeit with potential changes in their mechanisms. Protein synthesis-related domains, such as Ribosomal protein domains, were significantly enriched after treatment, implying enhanced protein synthesis and cellular repair. In contrast, oxidative stress-related domains, such as Metallothionein, showed a marked decrease in enrichment, indicating improved oxidative stress conditions. These findings suggest that the treatment may exert its effects by modulating key pathways, including metabolism, ferroptosis, immune responses, and cellular repair, to mitigate the pathological state induced by HFD (Fig. S3C, D).

The Go biological process enrichment analysis proved that GYJST treatment demonstrates significant protective effects against metabolic disorders induced by a high-fat diet. It regulates lipid metabolic imbalances by enhancing fatty acid β-oxidation, alleviates ferroptosis and related oxidative stress through iron binding and intracellular sequestering of iron ion, and modulates inflammatory responses to mitigate cell damage (Fig. 4E, F). In addition, it restores peroxisomal function, enhances antioxidant capacity, and reduces oxidative damage, collectively contributing to improved metabolic health.

Surprisingly, GSEA analysis reveals that GYJST can reverse the metabolic pathway alterations caused by HFD, including the biosynthesis of unsaturated fatty acids (Fig. 4G, H), peroxisome (Fig. 4I, J), and PPAR signaling pathways (Fig. 4K, L).

#### GYJST altered the liver metabolome

**Fig. 5:**
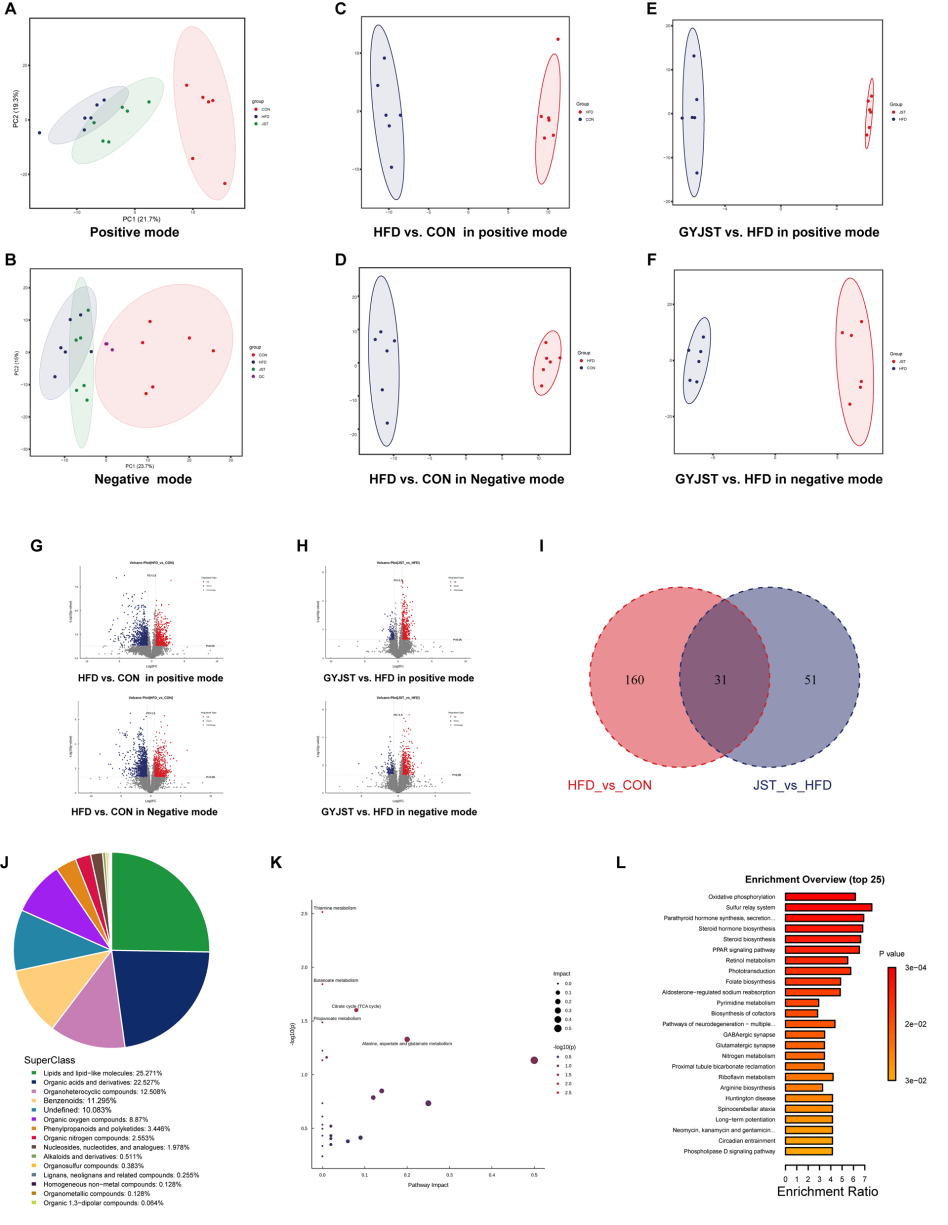
GYJST altered metabolite composition in HFD induced MAFLD. **A**, **B** Principal Component Analysis (PCA) of HFD versus CON and GYJST versus HFD. **C**–**F** Partial Least Squares Discriminant Analysis (PLS-DA) score plots of CON, HFD, and GYJST groups. **G**–**H** Volcano plots of differentially expressed metabolites (DMs) in HFD versus CON and GYJST versus HFD. **I** A Venn diagram of the DMs among the three groups. **J** Classification of 15 categories of endogenous DMs. **K** Pathway topology analysis in GYJST versus HFD group, and **L** Metabolite Set Enrichment Analysis (MSEA) of GYJST versus HFD group

The PCA results showed a clear distinction between CON, HFD and GYJST groups (Fig. 5A, B). To further identify the endogenous differential metabolites in mice liver intervened by GYJST, we applied a partial least squares discriminant analysis (PLS-DA) model. The liver samples from the three groups showed clear separation on the PLS-DA score plot in both positive and negative mode (Fig. 5C, F). After peak alignment and removal of missing values in more than 80% of samples, and based on the criteria of FDR < 0.05, and FC > 1.5 or < 1.5 (Fig. 5G, H), we screened and identified 242 endogenous different metabolites (DMs) in the liver (Table S6), including 191 metabolites in the HFD versus CON group, 82 metabolites in the GYJST versus HFD group, and 31 commonly regulated in three groups (Fig. 5I). The identified endogenous DMs belonged to 15 categories, with lipids and lipid − like molecules; organic acids and derivatives, organoheterocyclic compounds, benzenoids; organic oxygen compounds being the most regulated metabolites (Fig. 5J). Furthermore, A butterfly plot was utilized to intuitively illustrate the fold changes in significantly DMs in GYJST VS. HFD groups (Fig S4A, B). In the pathway topology analysis (Fig. 5K), the GYJST treatment group may enhance mitochondrial energy metabolism capacity by improving thiamine metabolism. It also regulates butanoate metabolism, which can enhance intestinal barrier function, reduce systemic inflammation, and modulate fatty acid metabolism, thereby alleviating lipid accumulation in adipose tissue. Citrate cycle were the major types of pathways regulated by GYJST (Fig. 5K). More importantly, metabolite set enrichment analysis (MSEA) were also introduced to explore the potential metabolic pathways (Fig. 5L). Interestingly, the PPAR signaling pathway was significantly enriched.

#### Integrated analysis of multi-omics experiments by network pharmacology

**Fig. 6:**
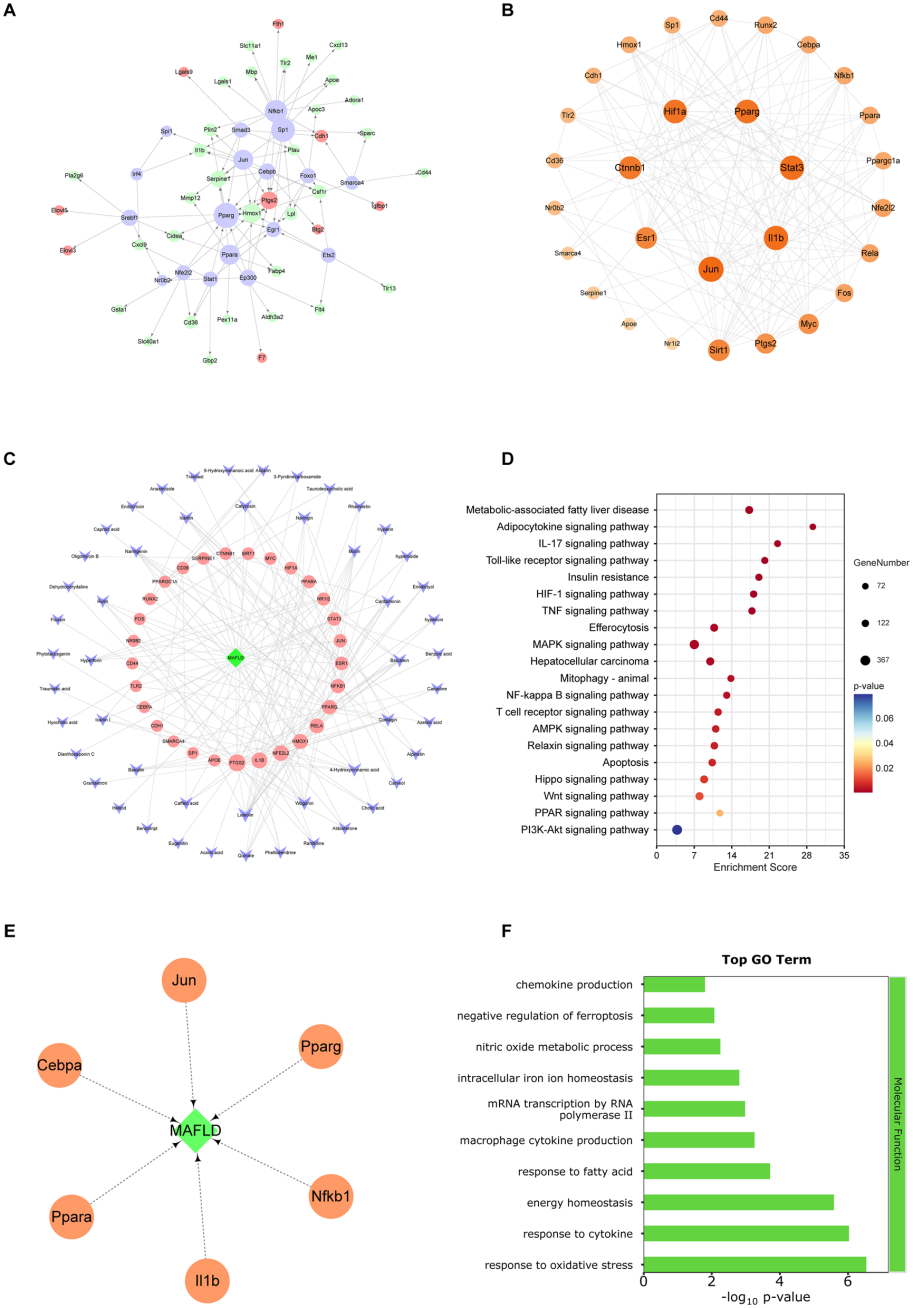
Integrative analysis of network pharmacology, transcriptomics and proteomics. **A** Gene Regulatory Network (GR Network) for GYJST Treatment. **B** PPI Network of GR Network and 148 Key Targets. **C** Network Construction of Ingredients, Candidate Targets, and MAFLD. **D**, **E** KEGG Pathway Analysis of Overlapping Targets. **F** GO Enrichment Analysis of Core Targets in Molecular Function (MF)

To further understand the underlying mechanisms for the effect of GYJST on MAFLD, we performed a network pharmacology study based on RNA-seq and proteomics results. Based on the mouse genome gene regulatory data in TRRUST database, we constructed the gene regulatory network (GR network) for DEGs and DEPs under GYJST treatment (GYJST vs. HFD) and then mapped mouse orthologs of GYJST putative targets on this network (Fig. 6A). Several hub nodes with higher degrees, such as PPARα, PPARg, COX2 and nuclear factor kappa B subunit 1 (NF-κB1), are key transcription factors in this network, potentially regulating the expression of other DEGs and DEPs. For the purpose of identifying potential therapeutic targets of GYJST, we mapped the GR network to 148 key targets related to compound targets and MAFLD. A total of 29 candidate targets were identified, and a PPI network was constructed using STRING and Cytoscape (Fig. 6B). To better elucidate the multi-component and multi-target mechanisms of traditional Chinese medicine, an interaction network linking ingredients, candidate targets, and MAFLD was constructed (Fig. 6C). Interestingly, the FerrDb database identified COX2 and nuclear factor (erythroid-derived 2)-like 2 (Nrf2/NFE2L2) as key ferroptosis markers, both exhibiting a higher degree in the network analysis. To explore the potential mechanisms, KEGG pathway analysis was conducted using the DAVID database. The results revealed that MAFLD was significantly enriched in relevant pathways (Fig. 6D). The corresponding targets are shown in Fig. 6E. To further investigate, GO enrichment analysis revealed that the Biological Process is associated with ferroptosis (Fig. 6F), which aligns with the findings from both proteomics and transcriptomics analyses.

#### GYJST inhibits system inflammation through NF-κB/COX-2 signaling axis in vivo

**Fig. 7:**
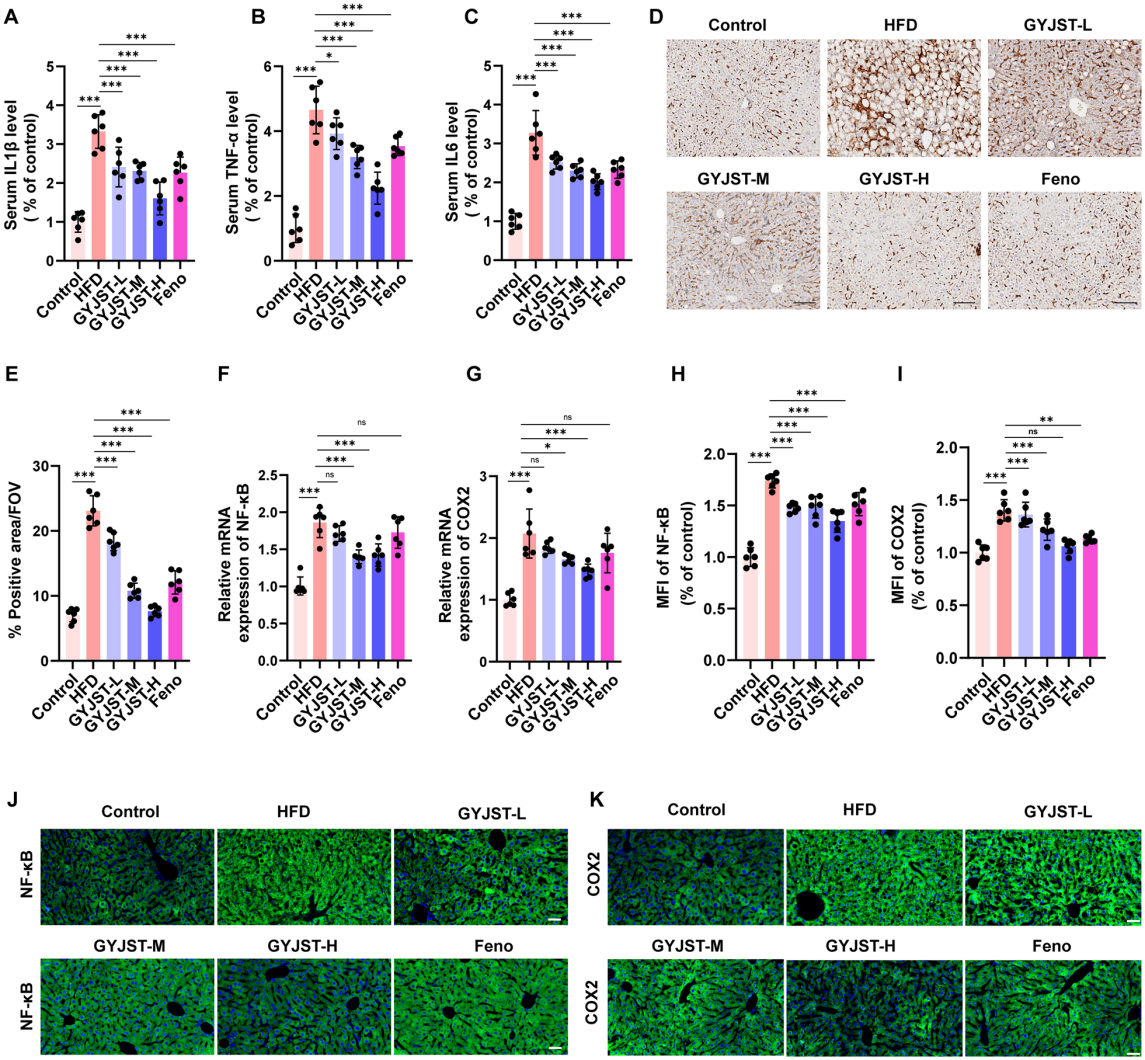
GYJST inhibits system inflammation through NF-κB/COX-2 signaling axis in vivo. **A**–**C** Effects of GYJST on liver IL-1β, TNF-α, IL-6. **D**, **E** IF was used to detect the inflammatory response of GYJST on liver (F4/80^+^) (scale bar: 100 μm, × 400). **F** Relative mRNA expression of NF-κB. **G** Relative mRNA expression of Cox2. **H**, **I** Quantification of the NF-κB and COX2 expression. **J**, **K** NF-κB and COX2 expression was measured using IF (scale bar = 50 μm). Statistical significance was calculated using one-way analysis of variance with Tukey’s multiple comparison test. n = 6, ^∗^p < 0.05, ^∗∗^p < 0.01 and ^∗∗∗^p < 0.001. ns, non-significant

Inflammation is a pivotal driver in the pathogenesis of fatty liver disease [21]. This study demonstrates that GYJST significantly ameliorates inflammation in HFD induced MAFLD by targeting the NF-κB/COX-2 signaling axis. Experimental results revealed that GYJST not only systemically reduced serum levels of pro-inflammatory cytokines (TNF-α, IL-1β, IL-6; Fig. 7A–C), but also markedly attenuated hepatic macrophage infiltration (F4/80^+^ cells; Fig. 7D, E), indicating its dual inhibitory effects on systemic and localized inflammatory responses. Mechanistic investigations guided by multi-omics analysis identified NF-κB and COX-2 as key targets, with functional validation showing that GYJST significantly suppressed hepatic mRNA expression of NF-κB and COX-2 (Fig. 7F, G), while concurrently blocking NF-κB p65 nuclear translocation and reducing COX-2 protein fluorescence intensity (Fig. 7H–K), thereby dual-targeting transcriptional activity and protein synthesis. Notably, GYJST exhibited superior efficacy compared to the conventional drug feno in alleviating hepatic inflammatory injury and inhibiting the NF-κB/COX-2 axis. These findings underscore GYJST’s unique polypharmacological mechanism, which synergistically disrupts the self-perpetuating inflammatory cycle by coupling NF-κB nuclear blockade with COX-2 expression downregulation, offering a novel therapeutic strategy distinct from existing treatments for MAFLD.

#### GYJST inhibits oxidative stress and lipid accumulation through Nrf2/PPARα/g signaling in vivo

**Fig. 8:**
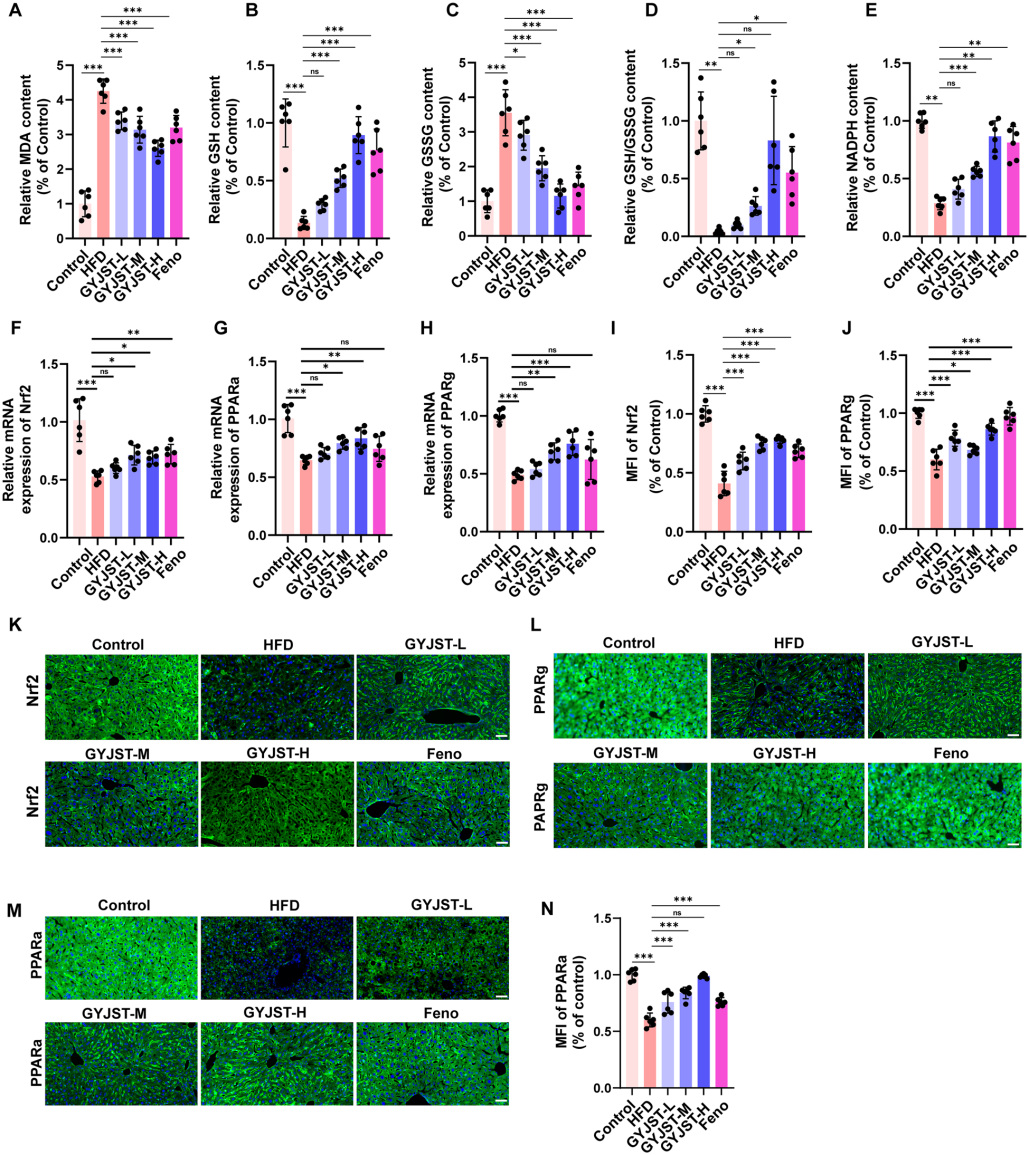
GYJST inhibits oxidative stress and lipid accumulation through Nrf2/PPARα/g pathway. **A**-**E** Effects of GYJST on liver MDA, GSH, GSSG, GSH/GSSG, NADPH in MAFLD mice. **F**-**H** Relative mRNA expression of Nrf2, PPAR a and PPAR g. **I**-**N** The Nrf2, PPARg and PPARa expression was measured using IF (scale bar = 50 μm). The results were expressed as mean ± SEM. Statistical significance was calculated using one-way analysis of variance with Tukey’s multiple comparison test. n = 6, ^∗^p < 0.05, ^∗∗^p < 0.01 and ^∗∗∗^p < 0.001. ns, non-significant.

Fat accumulation in the liver (lipotoxicity) leads to enhanced β-oxidation of free fatty acids (FFA), excessive reactive oxygen species (ROS) in mitochondria and peroxisomes, triggering a lipid peroxidation chain reaction and generating toxic products (MDA) which directly damage liver cell membranes and organelles [22]. In our research, GYJST significantly reduced the level of MDA, and upregulated the GSH/GSSG ratio and NADPH content, key molecules of the antioxidant system (Fig. 7A–E), to reconstruct redox homeostasis. After multi-omics analysis locked Nrf2 as the core regulatory target, qPCR results showed (Fig. 8F) that GYJST promoted Nrf2 mRNA transcription in a dose-dependent manner; immunofluorescence staining further confirmed (Fig. 8I, K) that it significantly enhanced the nuclear fluorescence intensity of Nrf2 protein, driving the expression of downstream antioxidant enzymes (HO-1, NQO1), thereby systematically improving the liver’s oxidative defense capacity. Furthermore, PPAR family plays an important role in lipid metabolic homeostasis [23]. Quantitative mRNA analysis demonstrated that GYJST dose-dependently enhanced the transcriptional activity of both PPARa and PPARg (Fig. 8G, H), while immunofluorescence staining also further revealed elevated nuclear translocation of their activated protein forms (Fig. 8I–N).

More importantly, it is worth noting that the activation of Nrf2 can further block the NF-κB signal [24], forming an “anti-oxidation-anti-inflammatory” positive feedback loop, thereby breaking the vicious interaction of oxidative damage, lipid metabolism disorders and inflammatory response, and providing a multidimensional intervention strategy for metabolic liver disease.

#### GYJST inhibits ferroptosis in vivo

**Fig. 9:**
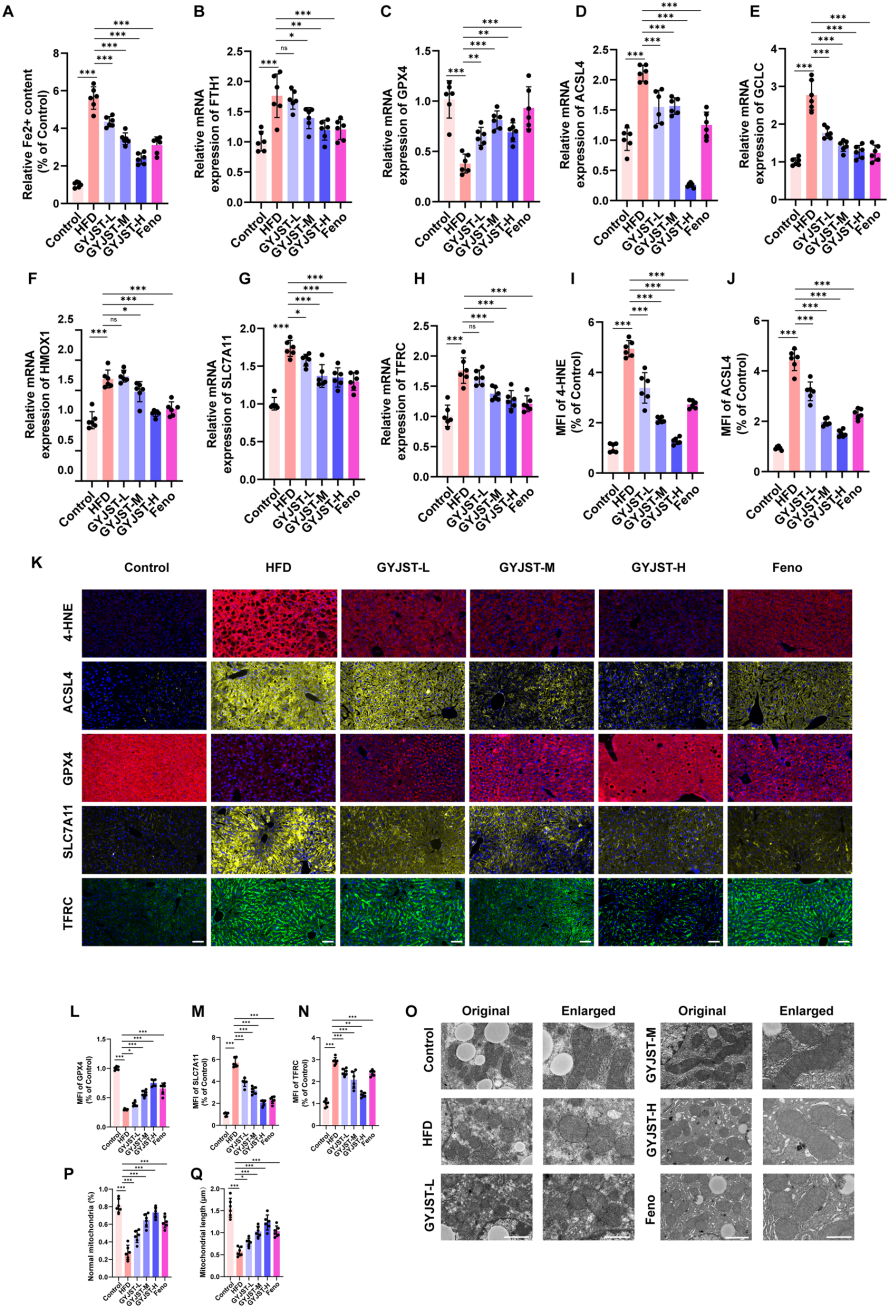
GYJST inhibits ferroptosis by regulating inflammation, oxidative stress, and lipid metabolism in vitro. **A** Serum Fe^2+^ concentration. **B**-**H** Relative mRNA expression of FTH1, GPX4, ACSL4, GCLC, HMOX1, SLC7A11, TFRC. **I**-**J** Quantification of mean fluorescence intensity of 4-HNE, and ACSL4. **K** Representative images of liver tissue stained with 4-HNE, ACSL4, Gpx4, SLC7A11, and TFRC (scale bar = 50 μm). **L**-**N** Quantification of mean fluorescence intensity of Gpx4, SLC7A11, and TFRC. **O** Representative transmission electron microscope (TEM) images of mice liver in three group Scale bar, 1 μm for original pictures and 0.5 μm for enlarged pictures. **P** Quantitative analysis of percentage of mitochondria with normal morphology in **O**. **Q** Quantitative analysis of average value of mitochondrial length (micrometers, μm) in **O**. Statistical significance was calculated using one-way analysis of variance with Tukey’s multiple comparison test. n = 6, ^∗^p < 0.05, ^∗∗^p < 0.01 and ^∗∗∗^p < 0.001. ns, non-significant

Through the analysis of core targets, we found that GYJST can regulate the ferroptosis process, which involved in iron accumulation, lipid peroxidation, and oxidative stress are usually involved. The lipid peroxidation and oxidative stress has been proved in previous research. Next, the Fe^2+^ level was presented in Fig. 9A. In addition, we performed qPCR for ferroptotic genes in liver tissue. We found that the expression of HMOX1, acyl-CoA synthetase long-chain family member 4 (ACSL4), FTH1, glutamate-cysteine ligase, catalytic subunit (GCLC) and solute carrier family 7 member 11 (SLC7A11) was upregulated, and the expression of GPX4 was downregulated (Fig. 7B–H). The immunofluorescence results showed an upregulation of the lipid peroxidation marker 4-HNE, ACSL4, SLC7A11 and TFRC, while GPX4 expression was downregulated (Fig. 7I–N). These findings suggest an increase in oxidative stress and ferroptosis-related processes, as indicated by the elevated lipid peroxidation and the altered expression of key regulatory proteins. Mitochondria play a key role in ferroptosis. We observed that after HFD, mitochondria in liver cells exhibited shrinkage, with condensed inner membranes and diminished cristae, as seen under transmission electron microscopy (Fig. 7O–Q).

#### GYJST inhibits inflammation, oxidative stress, lipid metabolism and ferroptosis in vitro

**Fig. 10:**
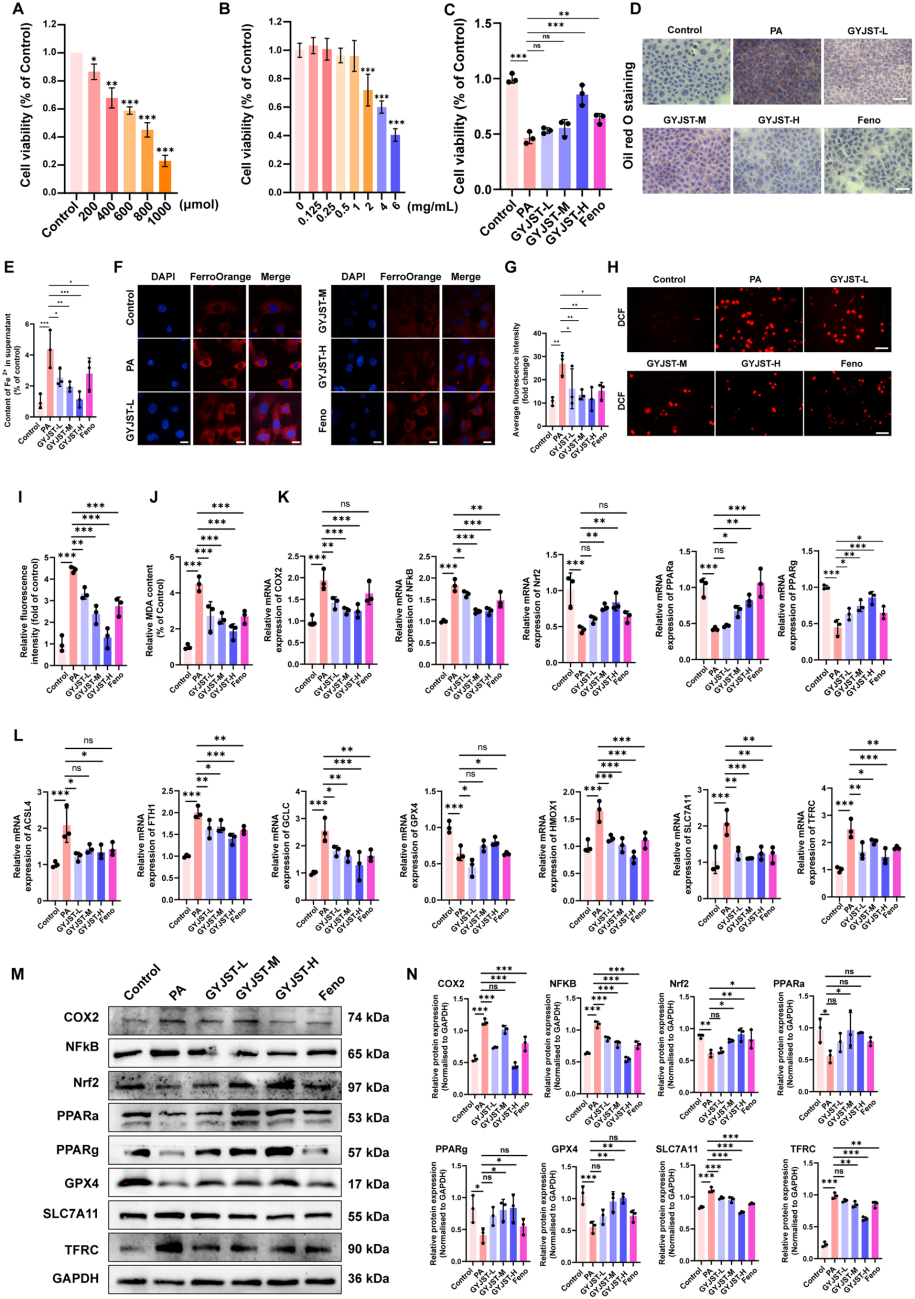
GYJST inhibits intracellular lipid deposition and ferroptosis in vitro. **A** Cell viability of AML12 cells treated with varying concentrations of palmitic acid (PA) was assessed using the CCK-8 assay. **B** Similarly, the CCK-8 assay was employed to evaluate the cell viability of AML12 cells exposed to different concentrations of GYJST. **C** The CCK-8 assay was employed to evaluate the cell viability of AML12 cells exposed to PA with different concentrations of GYJST and Feno. **D** Oil Red O staining in AML12 cells in different groups (magnification × 400, scale bar = 100 μm). **E** Relative ontent of Fe^2+^ in supernatant. **F**, **G** Intracellular ferrous iron (Fe^2+^) levels were detected using FerroOrange staining, followed by quantitative analysis (Scale bar, 10 μm). **H**, **I** ROS production was monitored using the H2DCFDA fluorescent probe (Scale bar, 100 μm), with subsequent quantification of fluorescence intensity. **J** Relative MDA content. **K**, **L** The relative mRNA expression levels of genes associated with inflammatory response, oxidative stress, lipid metabolism and ferroptosis were determined by qRT-PCR. **M**, **N** Protein expression levels of key markers of inflammatory response, oxidative stress, lipid metabolism and ferroptosis were analyzed by Western blotting. *p < 0.05, ***p < 0.001 and ns, non-significant, one-way ANOVA with Turkey’s multiple-comparison test. Data are represented as mean ± SD, n = 3

Palmitic acid (PA), a main biometabolite of saturated fatty acids, was used in vitro to gain further insights into the molecular mechanisms of lipotoxicity. Cell viability was measured by a concentration gradient to explore an appropriate concentration for PA treatment. The viability of AML12 cells treated with 400 μM PA decreased to 70%, compared to that of the control group (Fig. 10A). Therefore, in all subsequent experiments, we treated liver cell lines with 400 μM PA. To further assess the protective effects of GYJST [25, 26], different doses of GYJST lyophilized powder solution (0.125, 0.25, 0.5, 1, 2, 4 and 6 mg/mL) were filtrated and sterilized by 0.22 µm filter membrane. Then, a CCK-8 assay was performed to evaluate the cytotoxicity of GYJST. As shown in Fig. 10B, C, a concentration of 0.25, 0.5, 1 mg/mL was selected as a GYJST-L, GYJST-M, GYJST-H groups for the treatment. Besides, the Feno (50 μM) was supposed as the positive measures [27]. Staining of lipid droplets revealed that PA stimulation induced the production of numerous lipid droplets in the cytoplasm of AML12 liver cells, forming granular aggregates around the nucleus. Conversely, GYJST intervention notably reduced the formation of lipid droplets (Fig. 10D).

To further verify our previous results for HFD induced liver, we measured the concentration of ferrous iron in liver from the three groups. Our results demonstrated that PA exposure led to ferrous iron overload in both mouse liver cells and the culture supernatant. Treatment with GYJSTDS significantly mitigated this overload (Fig. 10E–G). ROS induced by ferrous iron overload are crucial for ferroptosis initiation. Using the H2DCFDA probe, we observed a significant increase in cellular ROS following PA exposure in AML12 cells, which was notably reduced by GYJSTDS. (Fig. 10H–I). MDA, as a biomarker of lipid peroxidation, was also assayed. PA exposure significantly enhanced lipid peroxidation in mouse testes (Fig. 10J).

Emerging evidence suggests that inflammation transcends its classical immunomodulatory roles to actively drive ferroptosis—a regulated cell death pathway characterized by iron-dependent lipid peroxidation [28]. As a well-known biomarker associated with ferroptosis and inflammation, the mRNA expression level of COX2 was significantly increased by twofold following treatment of AML12 liver cells with PA (Fig. 10K). The mRNA level of nuclear transcription factor NFKB was also upregulated (Fig. 10K), which could be inhibited by GYJST in a dose-dependent manner. More importantly, the mRNA expression of Nrf2, PPARa, PPARg, several important target genes of oxidative stress and lipid metabolism, was also upregulated (Fig. 10K). In addition, we performed qPCR for ferroptotic genes in AML12 cells. The result revealed that the expression of HMOX1, ACSL4, FTH1, GCLC, SLC7A11 and TFRC was upregulated, and the expression of GPX4 was downregulated. The treatment of GYJST could alleviate the damage trend (Fig. 10L). More importantly, the protein level of these core targets was presented in (Fig. 10M, N).

#### Nrf2 silencing inhibited the regulation of GYJST in the PPARg and GPX4 signaling pathway

**Fig. 11:**
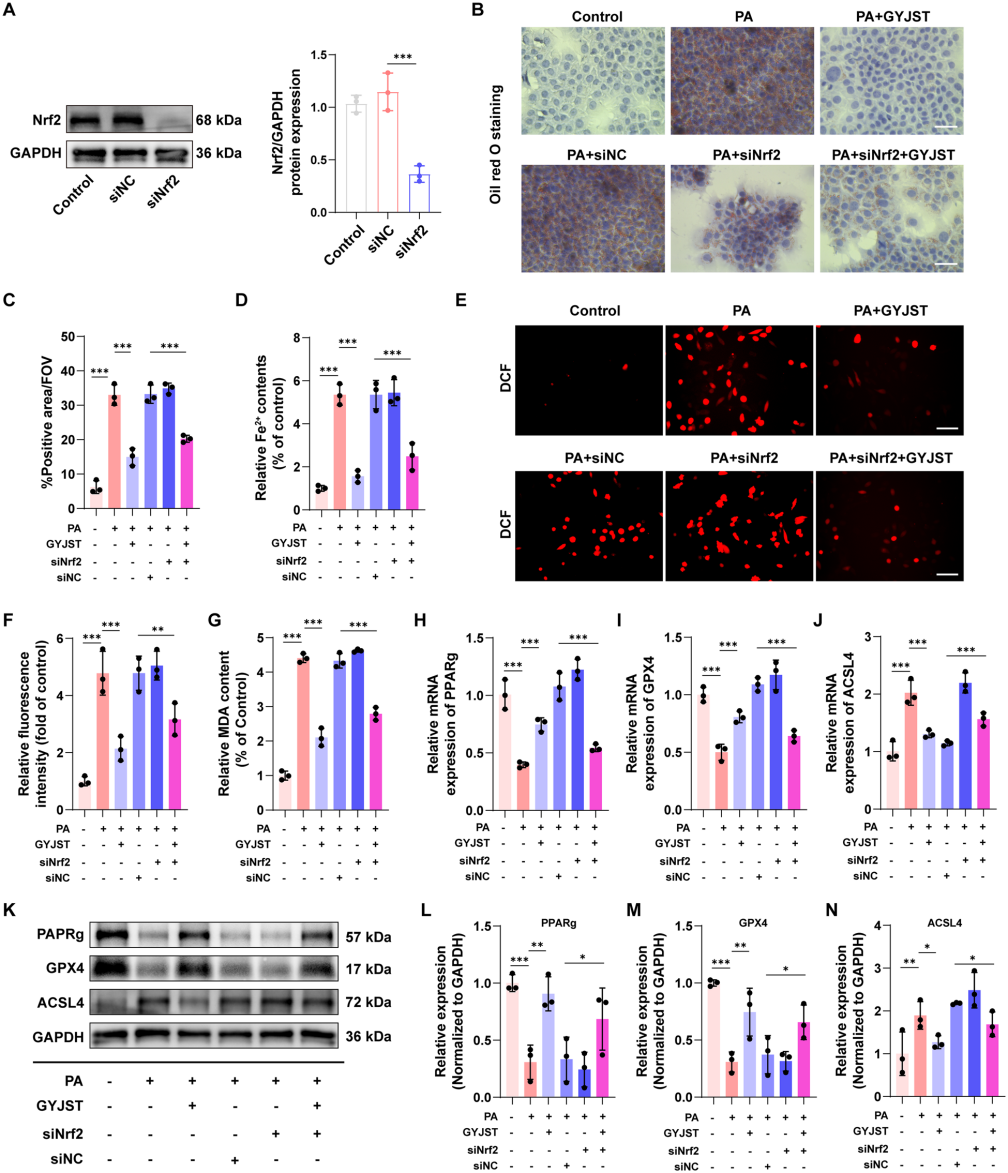
Nrf2 silencing inhibited the regulation of GYJST in the PPARg and GPX4 signaling pathway **A** Representative diagram and quantification analysis of Nrf2. Detection of Nrf2 protein was performed by Western blotting. **B** Oil Red O staining in AML12 cells in different groups (magnification × 400, scale bar = 100 μm). **C** Quantitative analysis of Oil Red O staining in (B). **D** Intracellular ferrous iron (Fe^2+^) levels were detected using Cell Ferrous Iron Colorimetric Assay Kit.** E**, **F** ROS production was monitored using the H2DCFDA fluorescent probe (Scale bar, 100 μm), with subsequent quantification of fluorescence intensity. **G** Relative MDA content in different groups. **H–J** The relative mRNA expression levels of PPARg, GPX4 and ACSL4 were determined by qRT-PCR. **K**–**N** Protein expression levels of PPARg, GPX4 and ACSL4 were analyzed by Western blotting. *p < 0.05, **p < 0.01 and ***p < 0.001, one-way ANOVA with Turkey’s multiple-comparison test. Data are represented as mean ± SD, n = 3

To investigate the role of Nrf2 in GYJST-mediated hepatoprotection, Nrf2 was silenced using siRNA. Western blot analysis revealed that AML12 cells transfected with siNrf2 exhibited the lowest Nrf2 protein level (Fig. 11A). Considering the dose-dependent effects of GYJST, a concentration of 1 mg/mL was applied in this part of the experiment. Oil Red O staining showed that PA treatment markedly increased lipid accumulation, while GYJST significantly reduced it. No difference was observed between the PA and PA + siNC groups, excluding transfection reagent effects. Lipid accumulation was further elevated in the PA + siNrf2 group, and while PA + siNrf2 + GYJST treatment partially reduced it, the effect was less pronounced than in the PA + GYJST group (Fig. 11B, C), suggesting that Nrf2 partly mediates GYJST’s regulation of lipid metabolism. Intracellular Fe^2^⁺ detection revealed that both PA + siNrf2 + GYJST and PA + GYJST reduced Fe^2^⁺ levels, with the strongest effect in the PA + GYJST group (Fig. 11D). DCFH-DA staining further showed that both treatments decreased ROS, though the antioxidant effect of GYJST was slightly diminished after Nrf2 silencing (Fig. 11E, F). Consistently, MDA levels were also reduced in both groups (Fig. 11G). Given the roles of PPARγ and GPX4 in lipid metabolism and ferroptosis, we examined their expression. Both genes were significantly upregulated at mRNA and protein levels in PA + siNrf2 + GYJST and PA + GYJST groups compared with PA (Fig. 11H, I, K, L and M). Conversely, ACSL4, a substrate provider for ferroptosis, was downregulated under both conditions (Fig. 11J, K and N). Together, these results indicate that GYJST alleviates oxidative stress, lipid accumulation, and ferroptosis in hepatocytes, with its protective effects partially mediated by Nrf2 signaling.

## Discussion

The HFD-induced MAFLD experimental model effectively replicates human clinical symptoms, characterized by liver lipid metabolic disorder and hyperlipidemia [29]. In this study, mice were fed an HFD for 12 weeks [30], followed by an 8-week treatment with GYJST. Notably, high doses of GYJST demonstrated a weight-reducing effect. Histopathological analysis revealed significant liver damage in HFD-fed mice, including hepatocyte steatosis and balloon degeneration, accompanied by elevated serum ALT and AST levels, consistent with previous findings [31]. GYJST treatment markedly ameliorated these pathological changes, restoring near-normal leaflet structures, reducing fat vacuoles, and promoting normal hepatocyte arrangement. Additionally, GYJST significantly lowered serum TC and TG levels, reduced lipid droplet deposition, and improved lipid homeostasis. Furthermore, fatty liver disease also related to inflammation and fibrosis [32], GYJST attenuated HFD-induced liver inflammation and fibrosis, as evidenced by decreased F4/80^+^ cell infiltration and NAS score. These findings collectively demonstrate that GYJST may alleviate HFD-induced hepatocyte damage in mice through its regulatory effects on inflammation and lipid metabolism [33].

During clinical application, the water extract of GYJST demonstrated potential for weight loss in obese patients and therapeutic effects on MAFLD. Using UHPLC-HRMS and network pharmacology analysis, we identified its multi-target mechanisms, including anti-inflammatory effects, oxidative stress modulation, lipid metabolism regulation, autophagy enhancement, and ferroptosis intervention, aligning with the multi-pathway nature of traditional Chinese medicine [34]. These findings strongly support further investigation of GYJST for MAFLD treatment.

To further evaluate the protective effects of GYJST on MAFLD, multi-omics technologies were employed, as they offer comprehensive insights into variations in genes, proteins, and metabolites associated with the disease [35]. Transcriptomics analysis revealed numerous differentially expressed genes (DEGs) between the model group and the GYJST group, including 113 down-regulated and 56 up-regulated genes. These DEGs were implicated in diverse biological processes, cellular components, and molecular functions, and were enriched in approximately 300 pathways. Notably, pathways related to iron ion homeostasis, oxidative stress, and inflammation were significantly enriched. Further analysis highlighted that the PPAR signaling pathway was prominently enriched in both groups, underscoring its potential role in the therapeutic mechanism of GYJST [36].

Transcriptomics primarily captures global variations in gene expression levels but often falls short of elucidating the underlying biological functions [37]. To address this limitation, we conducted a comprehensive proteomic analysis in this study. Our findings revealed 107 DEPs between the model and GYJST groups, comprising 58 up-regulated and 98 down-regulated proteins. GO analysis indicated that these DEPs were significantly enriched in various biological processes, cellular components, and molecular functions, particularly those associated with ferroptosis. Key terms included oxidoreductase activity, glutathione peroxidase activity, and lipid metabolic processes [38]. Furthermore, GSEA was conducted to investigate the effects of GYJST on metabolic processes at the protein level [39]. Notably, pathways including the biosynthesis of unsaturated fatty acids, peroxisome function, and PPAR signaling were significantly up-regulated in the HFD group, and GYJST treatment effectively reversed these alterations. These pathways regulate ferroptosis by modulating lipid metabolism and oxidative stress [40, 41]. Specifically, unsaturated fatty acid synthesis increases substrates for lipid peroxidation [42], peroxisomal dysfunction promotes ROS accumulation [43], and PPAR signaling influences ferroptosis through the regulation of metabolic and stress response pathways [44].

Based on the transcriptomics and proteomics predictions implicating lipid metabolism, oxidative stress, and inflammation in ferroptosis induction, we conducted metabolomics analysis for validation. Our metabolomics results revealed several DEMs in liver lysates between the GYJST and HFD groups, primarily associated with lipid metabolism pathways, including fatty acids, lipid molecules, and cholesterol metabolism. Furthermore, KEGG pathway analysis demonstrated significant involvement of the PPAR signaling pathway in MAFLD pathogenesis, consistent with our transcriptomic and proteomic findings.

To further elucidate the potential therapeutic targets of GYJST, we conducted an integrated network pharmacology analysis incorporating RNA-seq and proteomics data. Through comprehensive TRRUST database analysis [45], we identified Ppara, PPARg, COX2, and NF-kB1 as crucial transcription factors associated with the mechanism of GYJST. Furthermore, by constructing a “Component-Target-Disease” interaction network, we discovered Nrf2 as another key regulatory marker. Notably, Nrf2 has been widely recognized as a critical biomarker in ferroptosis regulation [46], suggesting its potential role in GYJST’s pharmacological effects. Therefore, we suppose that PPARa, PPARg, Cox2, NF-KB, and Nrf2, along with key ferroptosis markers such as GPX4, SLC7A11, ACSL4, and 4-HNE, collectively regulate HFD-induced hepatic ferroptosis through interconnected mechanisms. Under HFD conditions, the suppression of PPPARa activity disrupts lipid metabolism and oxidative stress responses, leading to increased lipid peroxidation, a hallmark of ferroptosis [47]. Similarly, PPARg dysfunction exacerbates oxidative damage and ferroptosis by impairing lipid storage and amplifying inflammatory responses. Concurrently, the upregulation of Cox2 promotes prostaglandin-mediated inflammation and oxidative stress, further driving ferroptosis progression. NF-kB, a central regulator of inflammation, is activated in this context, inducing pro-inflammatory factors and oxidative stress-related genes, which aggravate liver injury and ferroptosis [48]. Although Nrf2 typically serves as a protective factor by upregulating antioxidant genes (e.g., heme oxygenase-1 [HO-1] and NAD(P)H quinone oxidoreductase 1 [NQO1]) to counteract ferroptosis [49], its function is suppressed under HFD conditions, resulting in a compromised antioxidant defense system and accelerated ferroptosis. Furthermore, reduced GPX4 activity impairs lipid peroxidation clearance, while downregulated SLC7A11 decreases cystine uptake and glutathione synthesis, creating a permissive environment for ferroptosis [50]. The upregulation of ACSL4 enhances the esterification of polyunsaturated fatty acids, increasing cellular susceptibility to lipid peroxidation. Elevated levels of 4-HNE, a terminal product of lipid peroxidation, serve as a direct indicator of oxidative damage during ferroptosis [51]. Importantly, HFD-induced intracellular iron accumulation exacerbates these processes through the Fenton reaction, generating excessive ROS that amplify lipid peroxidation and cell membrane damage, ultimately driving ferroptosis. Together, these factors form a complex regulatory network that orchestrates HFD-induced hepatic ferroptosis. In subsequent studies, we validated these critical mechanisms through comprehensive in vivo and in vitro experiments.

Inflammation and oxidative stress often coexist with lipid accumulation [52], exacerbating adiposopathy and liver injury. As a physiological response to tissue damage, inflammation triggers the release of cytokines; however, excessive inflammation can drive the progression from simple fatty liver to non-alcoholic steatohepatitis (NASH) and even liver fibrosis [53]. Consistent with expectations, GYJST treatment significantly reduced levels of inflammatory cytokines (TNF-α, IL-1β, and IL-6) compared to the model group, demonstrating its anti-inflammatory and hepatoprotective effects. Moreover, GYJST treatment effectively reversed the upregulated mRNA expression of Cox2 and NF-κB induced by HFD, further supporting its anti-inflammatory properties.

Furthermore, hepatic oxidative stress, characterized by disrupted antioxidant systems, was observed in the HFD model group, as evidenced by elevated MDA and GSSG levels and decreased NADPH and GSH activities [54]. Notably, GYJST treatment effectively restored these oxidative markers to normal levels, demonstrating its ability to enhance antioxidant defenses and alleviate oxidative stress-induced liver damage. Additionally, GYJST significantly increased the mRNA expression of Nrf2, a key regulator of antioxidant responses, further supporting its role in mitigating oxidative stress.

GYJST effectively mitigates lipid accumulation, a key factor in reducing lipotoxicity and improving hepatocyte function. The intervention significantly reduced lipid droplet formation, as demonstrated by decreased Oil Red O staining intensity and lowered serum TG and TC levels. Furthermore, GYJST administration modulated key lipid metabolism regulators, notably reversing the mRNA expression of PPARα and PPARg. These findings collectively demonstrate GYJST’s potent lipid-lowering effects through multiple mechanisms.

Ferroptosis is an iron-dependent, non-apoptotic form of cell death characterized by the accumulation of lipid peroxides and the inhibition of GPX4 activity [55]. In recent years, the role of ferroptosis in various diseases, including MAFLD, has been increasingly recognized. In this study, in vivo experiments demonstrated that GYJST significantly reduced serum iron levels and downregulated the mRNA expression of ferroptosis-related genes, such as FTH1, ACSL4, GCLC, HMOX1, SLC7A11, and TFRC, while upregulating the mRNA expression of Nrf2 and GPX4. Immunofluorescence results further confirmed that GYJST reduced the levels of 4-HNE and TFRC and significantly increased GPX4 expression. Given the critical role of mitochondria in oxidative stress and ferroptosis, GYJST effectively mitigated mitochondrial dysfunction, thereby suppressing ferroptosis. In vitro experiments using the AML12 cell model showed that GYJST significantly ameliorated PA-induced steatosis, reduced intracellular iron content, and alleviated oxidative stress and inflammatory damage. Additionally, GYJST upregulated the protein expression of GPX4 and PPARg while downregulating the expression of ACSL4, PPARα, SLC7A11, and TFRC. Furthermore, GYJST improved PA-induced mitochondrial damage, further supporting its potential role in inhibiting ferroptosis and ameliorating metabolic disorders.

## Conclusion

**Fig. 12:**
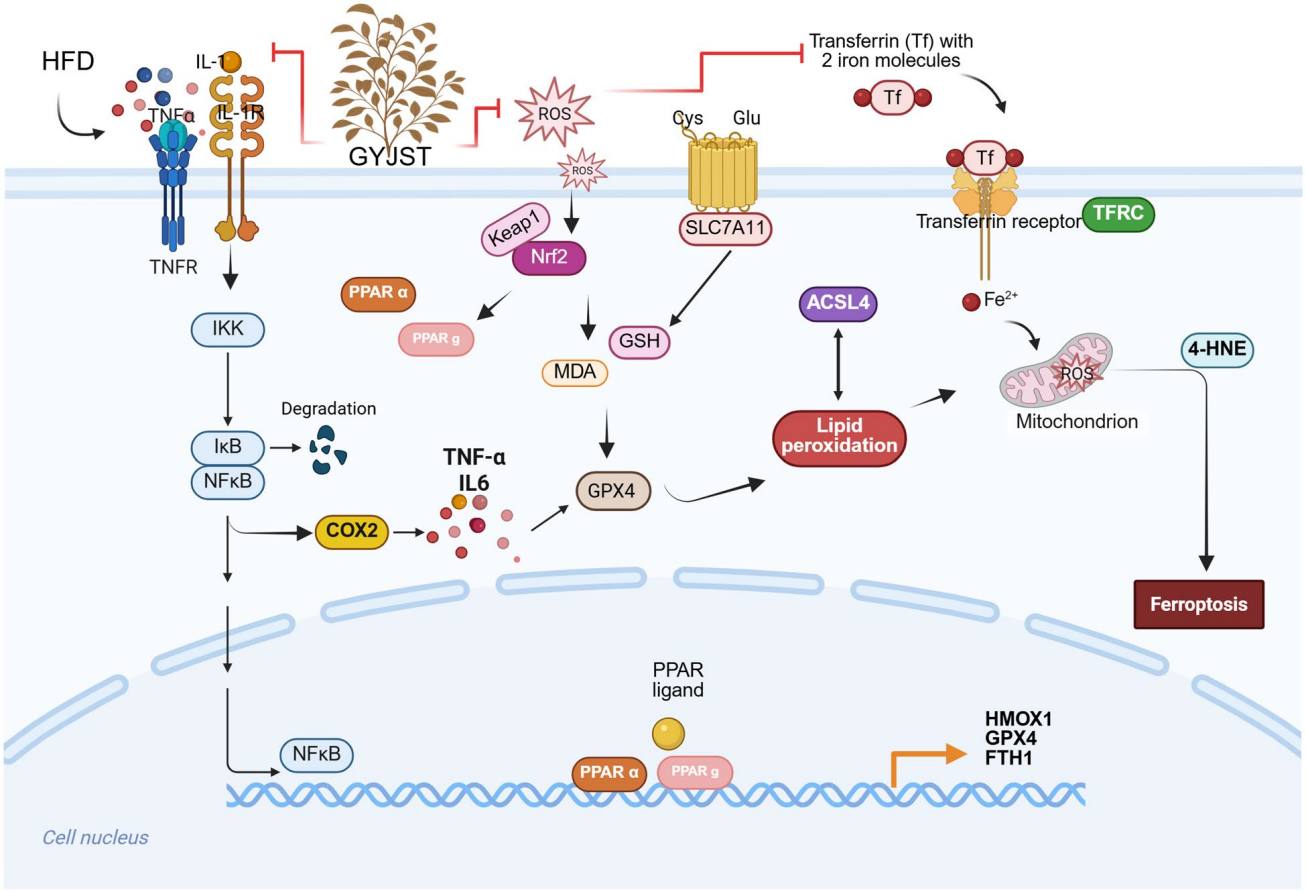
The mechanisms of anti-MAFLD effects of GYJST. GYJST suppresses systemic inflammation via the NF-κB/COX-2 signaling axis and alleviates oxidative stress and lipid accumulation through the Nrf2/PPARα/g pathway. In addition, GYJST regulates lipid metabolism by suppressing the ACSL4 and GPX4 pathways, and decreases intracellular iron accumulation, effectively attenuating ferroptosis and reducing liver injury and functional deterioration in MAFLD

In conclusion, this study demonstrates that GYJST effectively mitigates MAFLD progression by orchestrating the regulation of inflammatory cascades, oxidative stress, lipid metabolic disorders, and ferroptosis suppression (Fig. 12). Notably, the absence of dose–response analysis and potential interspecies differences between murine models and human pathophysiology may limit the direct translational relevance of these findings. Collectively, our results position GYJST as a multi-target therapeutic candidate for MAFLD, providing mechanistic insights into its interplay with ferroptosis and metabolic homeostasis. Further studies should prioritize pharmacokinetic profiling, clinical validation, and exploration of long-term efficacy to advance its therapeutic potential.

## Supplementary Information

Additional file 1.

Additional file 2.

## Data Availability

All data generated or analysed during this study are included in this published article.

## Abbreviations

MAFLD: Metabolic-associated fatty liver disease
NAFLD: Non-alcoholic fatty liver disease
MASH: Metabolic-associated steatohepatitis
ROS: Reactive oxygen species
DAMPs: Damage-associated molecular patterns
GPX4: Glutathione peroxidase 4
GYJST: Hypericum perforatum L
HFD: High-fat diet
siRNA: Small interfering RNA
ELISA: Enzyme-linked immunosorbent assay
IL-1β: Interleukin-1β
IL-6: Interleukin-6
TNF-α: Tumor Necrosis Factor alpha
PPARg: Peroxisome proliferator-activated receptor g
PPARα: Peroxisome proliferator-activated receptor alpha
TFRC/CD71: Transferrin receptor
4-HNE: 4 Hydroxynonenal
WB: Western blotting
UHPLC-HRMS: Ultrahigh-performance liquid chromatography-high resolution mass spectrometry
ESI: Electrospray ionization
DDA: Data-dependent acquisition
HCD: High-energy dissociation
CEs: Collision energies
H&E: Hematoxylin-eosin
TC: Total cholesterol
TG: Triglyceride
ALT: Alanine transaminase
AST: Aspartate aminotransferase
MDA: Malondialdehyde
TBA: Thiobarbituric acid
GSH: Glutathione
GSSG: Glutathione disulfide
NADPH: Nicotinamide adenine dinucleotide phosphate
NAS: NAFLD Activity Score
PFA: Paraformaldehyde
OCT: Optimal cutting temperature
TCMIP: Traditional Chinese Medicine
RIN: RNA Integrity Number
CCK-8: Cell Counting Kit-8
OD: Optical density
PI: Propidium iodine
DCFH-DA: 2′,7′-Dichlorodihydrofluorescein diacetate
GAPDH: Glyceraldehyde-3-phosphate dehydrogenase
RIPA: Radio immunoprecipitation assay
SDS-PAGE: Sodium dodecyl sulfate–polyacrylamide gel electrophoresis
PVDF: Polyvinylidene difluoride
SD: Standard deviation
NS: Non-significant
GO: Gene Ontology
PCA: Principal component analysis
DEGs: Differentially expressed genes
TNF: Tumor necrosis factor
IL-17: Interleukin-17
PPAR: Peroxisome proliferators activated receptor
GSEA: Gene set enrichment analysis
PPI: Protein-protein interaction
PTGS2/COX2: Prostaglandin-endoperoxide synthase 2
GSTM2: Glutathione S-transferase M2
DIA: Data independent acquisition
DEPs: Differentially expressed proteins
PLS-DA: Partial least squares discriminant analysis
DMs: Different metabolites
MSEA: Metabolite set enrichment analysis
NF-κB1: Nuclear factor kappa B subunit 1
Nrf2/NFE2L2: Nuclear factor (erythroid-derived 2)-like 2
GR network: Gene regulatory network
COX2: Cyclooxygenase-2
NF-kB: Nuclear factor kappa B
FFAs: Free fatty acids
HMOX1: Heme oxygenase (decycling) 1
FTH1: Ferritin heavy polypeptide 1
TEM: Transmission electron microscope
ACSL4: Acyl-CoA synthetase long-chain family member 4
GCLC: Glutamate-cysteine ligasecatalytic subunit
SLC7A11: Solute carrier family 7 member 11
PA: Palmitic acid
qRT-PCR: Quantitative real-time PCR
HO-1: Heme oxygenase-1
NQO1: NAD(P)H quinone oxidoreductase 1
NASH: Non-alcoholic steatohepatitis
IF: Immunofluorescence
MF: Molecular function
Fe^2+^: Ferrous iron
