# Supplementary material for: Hypericum perforatum L. extract alleviates metabolic-associated fatty liver disease through inflammation, lipid metabolism and ferroptosis modulation: a multi-omics perspective
Source: Chin Med. 2025 Dec 3;20:210. doi: 10.1186/s13020-025-01248-1 (PMC12673736; doi:10.1186/s13020-025-01248-1)
Supplement: Supplementary file 1 — Additional file 1. [file 13020_2025_1248_MOESM1_ESM.docx]

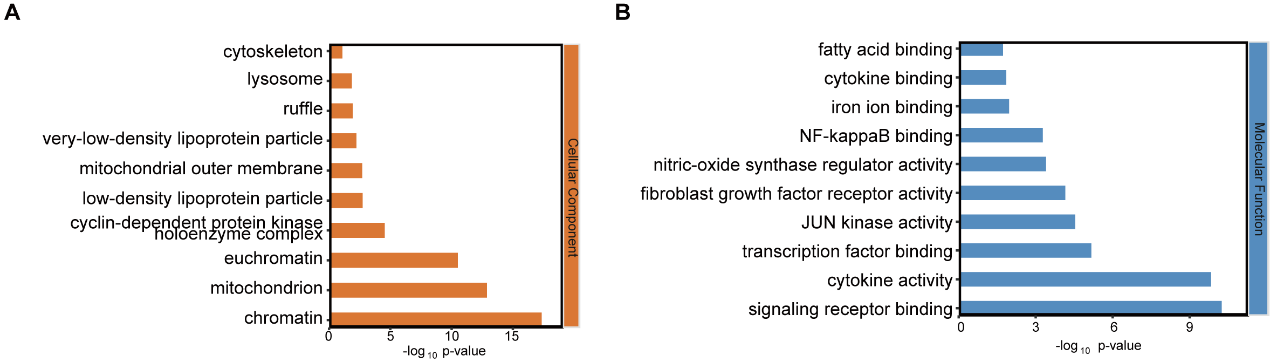


**Fig. S1. GO enrichment analysis of core targets in cellular component (CC) and molecular function (MF).**


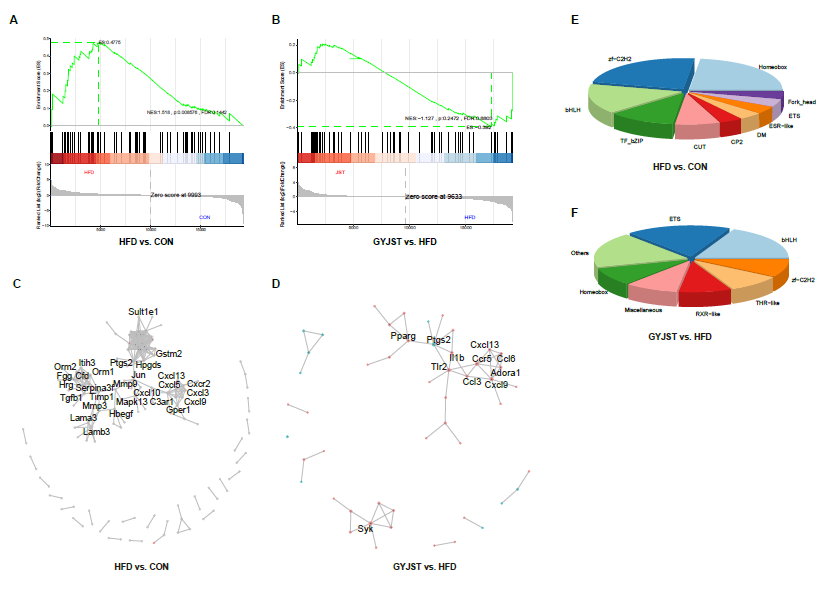


**Fig. S2. Transcriptomics analysis.** (A-B) Gene set enrichment analysis (GSEA) for the biosynthesis of PPAR signaling pathways in HFD *vs.* CON and GYJST *vs.* HFD. (C-D) Protein-Protein Interaction (PPI) Network of HFD *vs.* CON and GYJST *vs.* HFD. (E-F) Transcription factor annotations for degs in HFD *vs.* CON and GYJST *vs.* HFD.


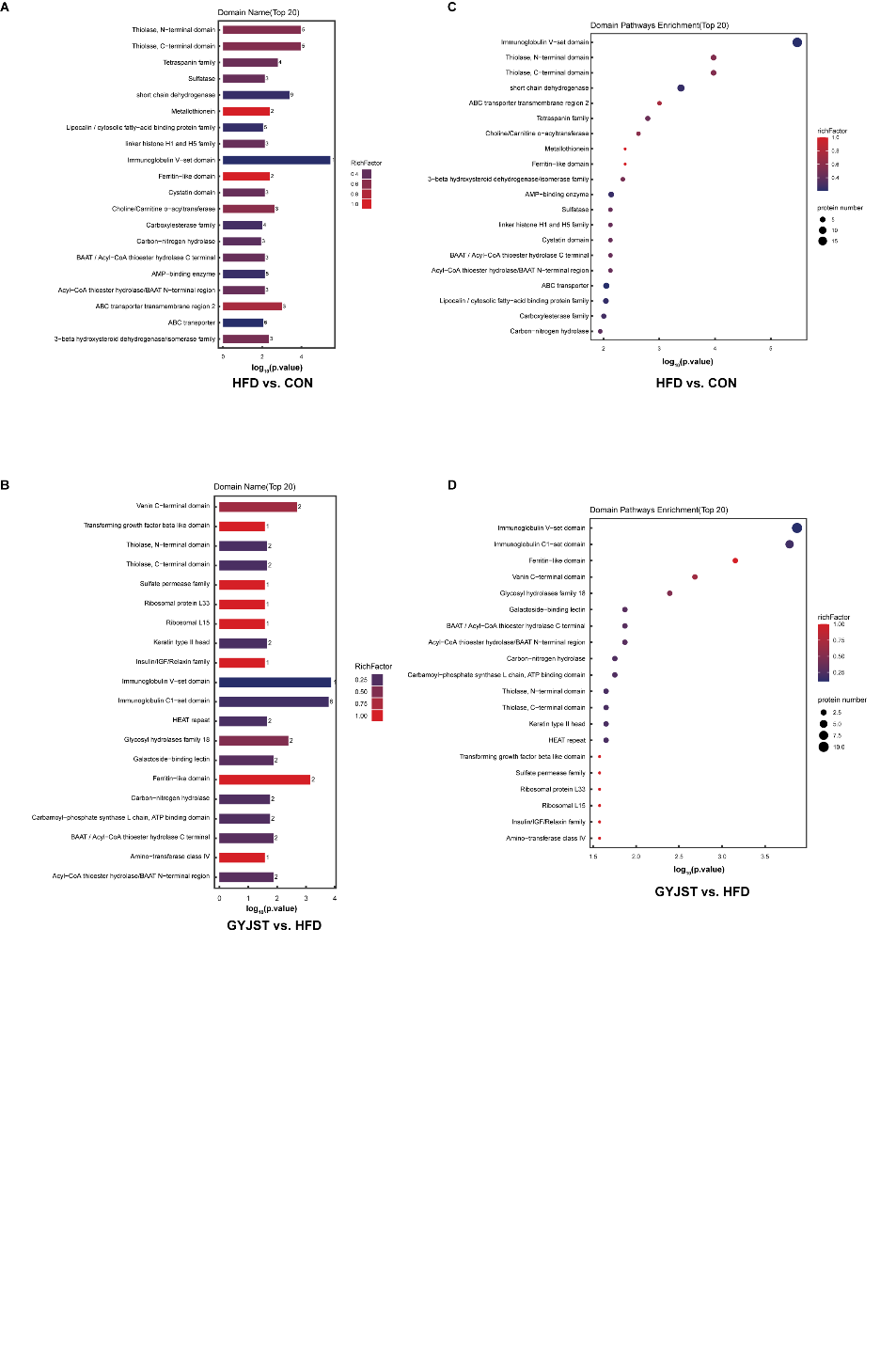


**Fig. S3. Proteomics analysis.** (A-B) Top 20 domain names of different proteins in HFD vs CON and GYJST vs HFD. (C-D) Pfam domain enrichment analysis of different proteins in HFD vs CON and GYJST *vs.* HFD.


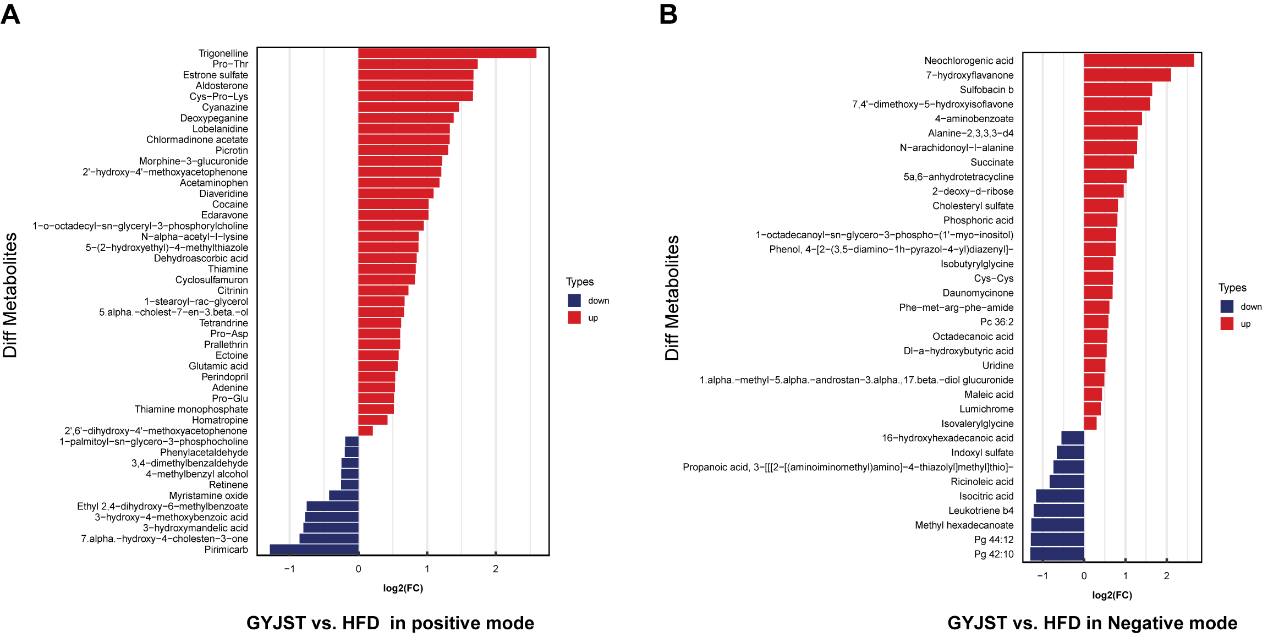


**Fig. S4. Metabolome analysis.** (A-B) Butterfly diagram analysis of different metabolites of HFD vs CON in positive models and negative models.
